# Downsizing macrocycles in melanocortin peptide analogues: thioether and lactam cyclization strategies to modulate *h*MC3/4R agonism and antagonism

**DOI:** 10.1039/d6md00726k

**Published:** 2026-10-21

**Authors:** Rosa Bellavita, Luis E. Gimenez, Savannah Y. Williams, Ida Boccino, Gennaro M. Bailo, Roger D. Cone, Paolo Grieco, Francesco Merlino

**Affiliations:** a Department of Pharmacy, University of Naples Federico II Naples 80131 Italy francesco.merlino@unina.it; b Life Sciences Institute, University of Michigan Ann Arbor MI 48109 USA; c Department of Molecular and Integrative Physiology, University of Michigan Ann Arbor MI 48109 USA; d Department of Molecular, Cellular, and Developmental Biology, University of Michigan Ann Arbor MI 48109 USA

## Abstract

Stabilizing β-turn geometry through macrocyclization has been a key determinant of receptor selectivity and functional activity in melanocortin peptide ligands. In addition to conformational constraint, macrocycle size plays a critical role in modulating receptor recognition. In this study, we report a novel library of downsized **MT-II** and **SHU-9119** analogues featuring 20- and 19-membered macrocycles, generated through lactam and thioether cyclization strategies while preserving the canonical His–dPhe/dNal(2)–Arg–Trp pharmacophore. Functional evaluation revealed that these reduced macrocycles retained activity at the human melanocortin-3 and -4 receptors (*h*MC3R and *h*MC4R), with key structural elements governing receptor preference and agonist *versus* antagonist behavior within this receptor subset. Three new *h*MC4R agonists were identified: the lactam-linked peptides 6 and 10 and the thioether-linked peptide 14. In addition, the thioether-linked peptide 15 exhibited weak partial agonist activity and functional antagonism at *h*MC4R. Notably, peptide 14 showed subnanomolar *h*MC4R agonist potency (pEC_50_ = 9.55) and 84-fold functional preference for *h*MC4R over *h*MC3R. These findings identify the 20-membered thioether-containing architecture as particularly favourable for achieving potent *h*MC4R agonism and enhanced functional preference over *h*MC3R.

## Abstract 2

Reducing macrocycle size while preserving the melanocortin pharmacophore provides a means to tune functional activity and receptor preference at *h*MC3R and *h*MC4R.

## Graphical abstract

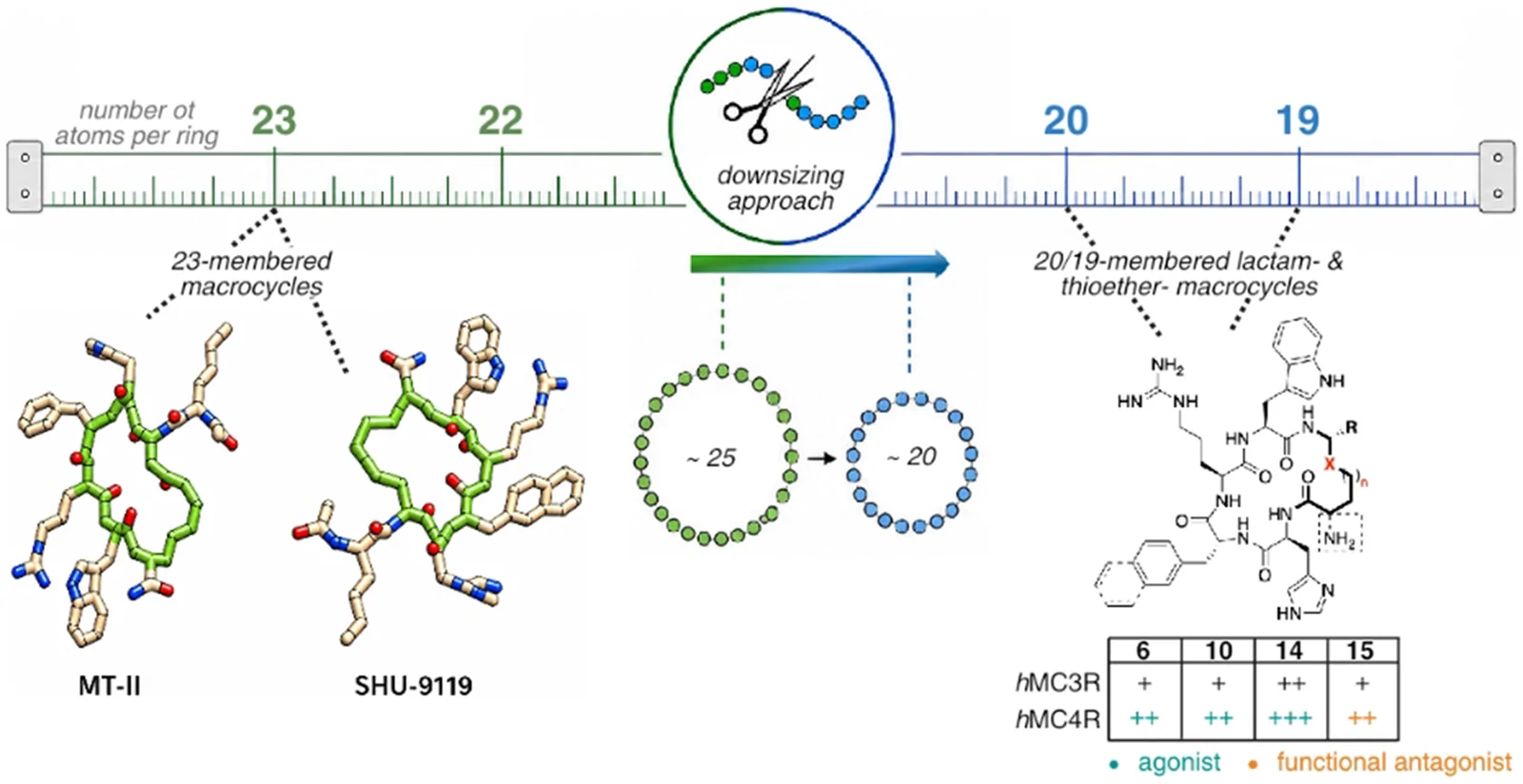

## Introduction

Peptides targeting the melanocortin receptor system have attracted considerable attention due to their involvement in fundamental physiological processes, including pigmentation, energy homeostasis, feeding behavior, and inflammation.^1–5^ The melanocortin system consists of five G protein-coupled receptors (MC1R–MC5R) activated by endogenous peptides derived from pro-opiomelanocortin (POMC).^6,7^ Among these, α-melanocyte-stimulating hormone (α-MSH), adrenocorticotropic hormone (ACTH), and related melanocortins function as key agonists, exhibiting distinct but overlapping affinities for different receptor subtypes. *h*MC1R preferentially responds to α-MSH over ACTH, β-MSH, and γ-MSH and is primarily associated with skin pigmentation and anti-inflammatory responses.^8^ MC2R, the canonical ACTH receptor, is essential for adrenal steroidogenesis and mediates the physiological stress response.^9^ MC5R, which is activated by α-MSH > ACTH, β-MSH, and γ-MSH, is mainly linked to exocrine gland function.^10^ In contrast, MC3R and MC4R are central components of the leptin–melanocortin axis and represent closely related yet functionally divergent regulators of energy balance, appetite, and metabolic homeostasis.^11–14^ MC3R preferentially responds to γ-MSH over α-MSH and β-MSH and has a primary role as a negative regulator of MC4R neurons *via* its presynaptic expression on the GABAergic AgRP neurons, and also regulates communication between nutritional status and reproductive function, whereas MC4R, which displays higher affinity for β-MSH followed by α-MSH and γ-MSH, functions as a dominant regulator of energy expenditure.^15–20^ Beyond metabolic control, MC4R signaling has also been implicated in sexual function and cardiovascular homeostasis.^21^ From a pharmacological perspective, the functional divergence between MC3R and MC4R is particularly intriguing, as it arises not only from differences in ligand preference but also from receptor-specific regulatory mechanisms that shape the signaling output.^22^ Within the leptin–melanocortin pathway, these receptors exhibit distinct phenotypic consequences, reflecting differential protein–protein interactions, membrane trafficking, and signal modulation.^14^ Consistent with this complexity, disruption of the POMC–melanocortin system, through POMC deficiency or loss-of-function mutations in MC4R, leads to profound dysregulation of energy homeostasis and is strongly associated with severe early-onset obesity and related metabolic disorders.^23^

Endogenous melanocortins exhibit limited receptor selectivity and display overlapping binding affinities across MC receptor subtypes. This limited specificity poses a significant challenge for therapeutic exploitation, as the activation of non-target receptors may lead to undesirable physiological effects. Nevertheless, the development of receptor-selective melanocortin analogues holds substantial therapeutic promise across a broad range of indications. In particular, MC4R-selective agonists are actively pursued for treating obesity and metabolic disorders, whereas MC3R-selective ligands may provide unique opportunities for addressing metabolic syndrome, reproductive function, cachexia and inflammatory diseases.^24,25^ Simultaneously, recent studies indicate that carefully balanced dual MC3R/MC4R agonism may represent an alternative therapeutic strategy for obesity by exploiting complementary functions of the two receptors.^26^ Therefore, achieving subtype selectivity within the melanocortin receptor family is critical, given both the extensive physiological roles governed by these receptors and the potential liabilities associated with off-target signaling.^27^ In this context, the design of peptide analogues with tailored receptor selectivity toward specific melanocortin receptors represents a promising strategy to overcome these challenges, enabling the advancement of therapeutic applications while mitigating the risk of off-target effects and improving our understanding of receptor-specific signaling pathways. A central approach to enhancing receptor selectivity involves developing conformationally constrained cyclic peptides.^28^ Peptide cyclization can improve receptor binding affinity by stabilizing the bioactive conformation and limiting flexibility. Moreover, cyclization reduces the overall entropy loss associated with receptor binding, thereby frequently increasing potency and selectivity. Consistent with these advantages, a wide range of cyclic melanocortin analogues have been reported.^29,30^ Based on the core tetrapeptide sequence His–Phe–Arg–Trp of α-MSH, which is highly conserved and essential for melanocortin receptor activation, targeted modifications of the α-MSH scaffold have proven particularly successful in the development of representative cyclic derivatives. In the 1980s, seminal analogues such as **MT-II** (1, Fig. 1), a potent but nonselective agonist at *h*MC1R, *h*MC3R, *h*MC4R, and *h*MC5R, and **SHU-9119** (2, Fig. 1), a nonselective antagonist with high affinity for *h*MC3R and *h*MC4R while acting as an agonist at *h*MC1R and *h*MC5R, were discovered.^31–33^ These compounds established cyclic melanocortin peptides as powerful tools for probing receptor function and structure–activity relationships (SAR). Subsequently, building on the **MT-II** scaffold, *bremelanotide* (PT-141) (3, Fig. 1) was developed by Palatin Technologies and marketed under the trade name Vyleesi® as a self-administered, on-demand subcutaneous therapy for female sexual dysfunction.^34^*Bremelanotide* is a cyclic lactam heptapeptide derived from **MT-II**, in which the C-terminal amide is replaced by a carboxylate. This agonist displays functional selectivity toward *h*MC3R and *h*MC4R (EC_50_ ≈ 0.25 nM) in the central nervous system, where it modulates sexual desire and arousal.^35^ In 2019, the FDA approved *bremelanotide* for the treatment of hypoactive sexual desire disorder (HSDD) in premenopausal women.^36^ Currently, the drug is commercially available only in the United States, and approval in Europe and other regions is still pending. Recently, *setmelanotide* (4, Fig. 1) was approved in 2020 as an anti-obesity agent.^37^*Setmelanotide* (Imcivree®) is a cyclic octapeptide exhibiting high potency toward two centrally acting melanocortin receptors, with EC_50_ values of 2.3 nM at MC3R and 0.4 nM at MC4R. This synthetic peptide was approved by the FDA for the treatment of obesity resulting from rare genetic defects in POMC, leptin receptor (LEPR), or proprotein convertase subtilisin/kexin type 1 (PCSK1), and resulting from hypothalamic damage in syndromes such as craniopharyngioma.^37^

**Fig. 1:**
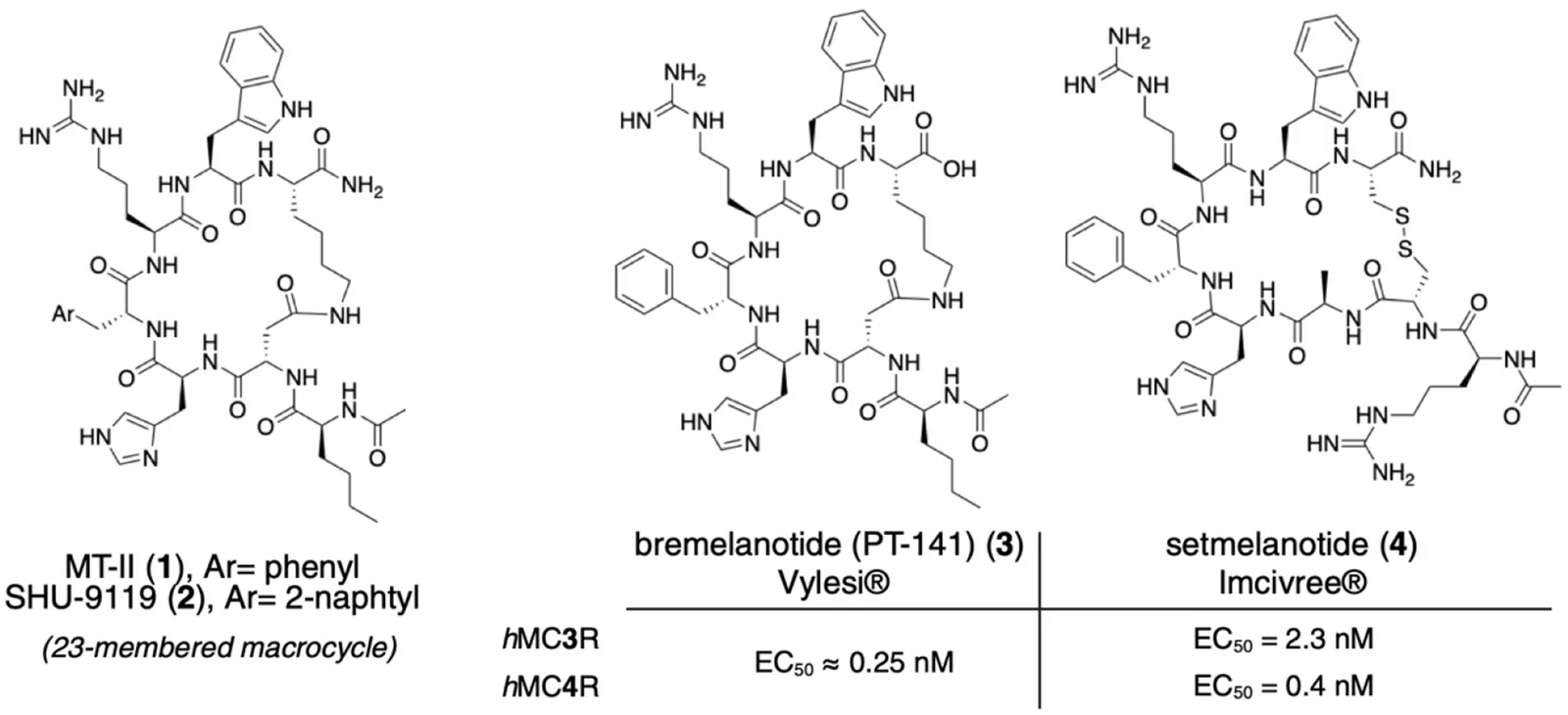
Chemical structures of representative cyclic melanocortin peptide analogues derived from the α-MSH pharmacophore. **MT-II** (1) and **SHU-9119** (2) are early macrocyclic ligands featuring a 23-membered ring and displaying non-selective agonist and antagonist profiles, respectively. *Bremelanotide* (PT-141, 3) is a cyclic lactam heptapeptide with functional selectivity toward *h*MC3R and *h*MC4R, whereas *setmelanotide* (4) is a cyclic octapeptide approved for the treatment of rare genetic forms of obesity, exhibiting high potency at *h*MC4R and *h*MC3R.

In 2008, building on the **MT-II** scaffold and aiming to improve both pharmacokinetic and pharmacodynamic properties, Grieco *et al.* reported the development of **PG10N** (5, Fig. 2).^38^ This cyclic peptide analogue is characterized by a macrocyclization strategy based on an alkylthio–aryl bridge, introduced *via* nucleophilic aromatic substitution (S_N_Ar). Compared with **MT-II**, **PG10N** shows marked structural differences, most notably a reduction in ring size from 23-membered to 19-membered. The incorporation of the alkylthio–aryl linkage resulted in a more conformationally constrained scaffold, which contributed to improved receptor binding and selectivity across the melanocortin receptors. Pharmacological evaluation revealed that **PG10N** exhibited high affinity and efficacy at *h*MC1R (IC_50_ = 3.7 nM; EC_50_ = 2.0 nM), while showing reduced functional inhibitory potency at *h*MC3R (IC_50_ = 40 nM) and *h*MC4R (IC_50_ = 47 nM), albeit with comparable potency (EC_50_ ≈ 2 nM). In contrast, functional inhibitory potency at *h*MC5R was significantly weaker (IC_50_ = 190 nM; EC_50_ = 44 nM), indicating reduced receptor potency.^38^ Overall, **PG10N** emerged as one of the most effective constrained melanocortin analogues reported at the time, despite displaying a narrower activity profile relative to broader-spectrum agonists.

**Fig. 2:**
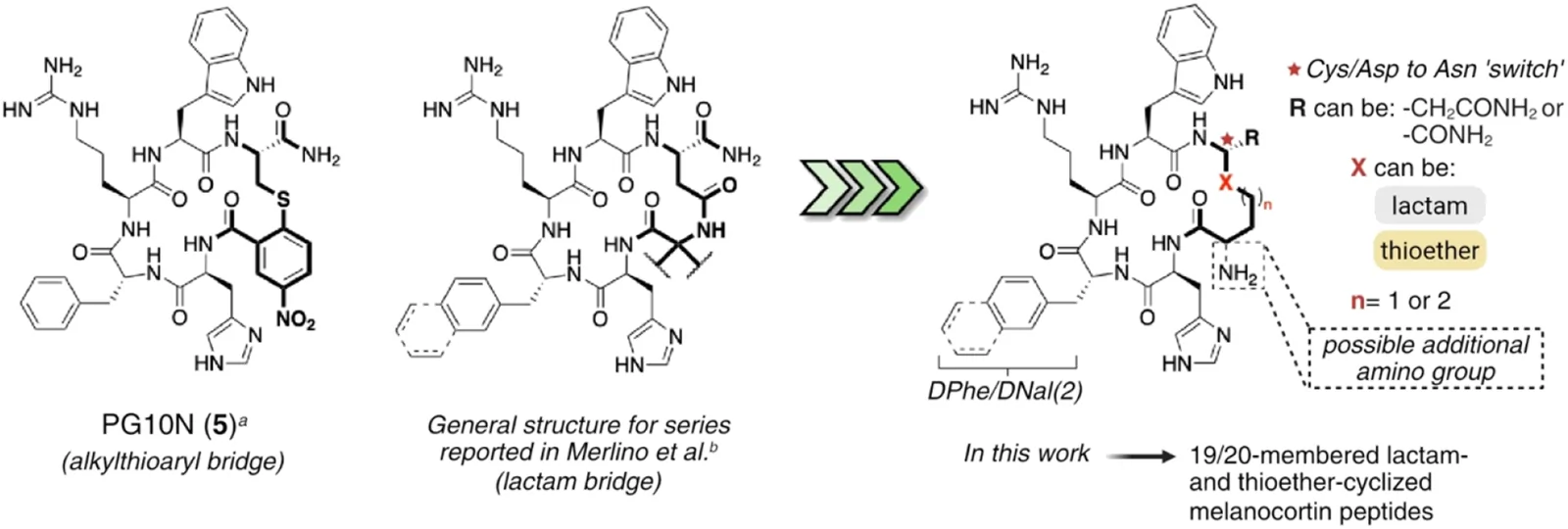
Schematic overview of the design of downsized cyclic melanocortin peptide analogues. Previously reported **PG10N** (5), featuring an alkylthioaryl bridge, and lactam-cyclized scaffolds were used as reference structures. The present study explores macrocycle downsizing *via* lactam- and thioether-based cyclization strategies to afford 19- and 20-membered rings, while systematically varying cyclization topology, linker length (*n* = 1 or 2), and key pharmacophoric residues [dPhe/dNal(2)]. ^a^See ref. 38; ^b^see ref. 40.

Subsequently, Yue *et al.* revisited **PG10N** to explore the versatility of the S_N_Ar-based macrocyclization strategy by further functionalizing the nitroaryl moiety.^39^ In particular, the nitro group was employed as a synthetic handle or *a priori* substituted with cyano or ethynyl groups, for post-cyclization derivatization into aniline, fluorescent labeling, amidinylation, and glycosylation. In combination with ring expansion achieved by insertion of an alanine residue, several of these modifications led to the identification of potent and selective *h*MC4R agonists. Notably, replacement of the nitro group with an amine (*e.g.*, compound 10; pEC_50_ = 8.80 ± 0.60, p*K*_i_ = 8.52 ± 0.15), an amidine (*e.g.*, compound 11; pEC_50_ = 9.56 ± 0.12, p*K*_i_ = 9.76 ± 0.19) or a fluorescein (FITC) label (*e.g.*, compound 12; pEC_50_ = 9.00 ± 0.02) resulted in marked improvements in *h*MC4R potency and functional selectivity.^39^

Maintaining the same 19-membered ring size as **PG10N**, additional cyclic lactam analogues were subsequently designed featuring side-chain-to-tail cyclization and the incorporation of conformationally constrained amino acids. In particular, analogues containing 2-aminoisobutyric acid (Aib) and related α,α-dialkylated residues in the ring-closing segment were investigated by some of us for their interactions with melanocortin receptors.^39^ Several compounds displayed unprecedented activity and selectivity profiles, including selective agonists at *h*MC3R (*e.g.*, compound 8; IC_50_ = 10 nM; EC_50_ = 30 nM), as well as ligands exhibiting mixed functional behaviour, acting as partial agonists or antagonists depending on the receptor subtype.^40^

These findings further demonstrated the effectiveness of constrained melanocortin scaffolds featuring 19- and 20-membered macrocycles in modulating receptor selectivity and signalling outcomes. More broadly, the incorporation of chemical constraints such as disulfide bridges, lactam linkages, or peptide stapling has emerged as a powerful strategy to impose structural rigidity and stabilizing conformations that favor selective receptor engagement.^41^ Building on prior evidence that 19-membered lactam macrocycles retain activity at *h*MC3R and *h*MC4R, we investigated how cyclization chemistry (lactam *vs.* thioether), macrocycle size, and ring topology influence receptor preference and activity at *h*MC3R and *h*MC4R. We systematically compared 19- and 20-membered lactam and thioether macrocycles featuring ‘*head-to-side-chain*’ and ‘*head-to-tail*’ cyclization topologies. Specifically, by integrating chemical synthesis with functional *in vitro* assays, this study explores the structural determinants governing functional preference between *h*MC3R and *h*MC4R and the associated functional outcomes.

## Results and discussion

### Design

The rational design of novel cyclic melanocortin peptide analogues was guided by previous SAR studies, which identified three key determinants for receptor subtype selectivity: i) preservation of the His–dPhe/dNal(2)–Arg–Trp pharmacophoric core, ii) reduction of macrocycle size, and iii) preservation of the bioactive conformation. Previous studies have demonstrated that downsizing the macrocycle from 23 members, as in **MT-II** and **SHU-9119**, to 19 members, as exemplified by the thioether-bridged peptide **PG10N**, led to improved pharmacological profiles, including enhanced receptor selectivity and functional modulation.^38^ Accordingly, the present design strategy was inspired by **PG10N** and subsequent derivatives reported by Yue *et al.*,^38,39^ which highlighted the nitro group of **PG10N** as an additional site amenable to functionalization and potentially relevant for modulating interactions with melanocortin receptors, particularly *h*MC3R and *h*MC4R (Fig. 2). With respect to the macrocyclization motif, our design was informed by two complementary precedents. On one hand, we drew inspiration from the lactam bridges employed in downsized analogues previously reported by some of us (*e.g.*, compound 8 in Merlino *et al.*) (peptides 6–13).^40^ On the other hand, we considered the alkyl thioether bridge featured in **PG10N** and related analogues reported by Yue *et al.* (*e.g.*, compounds 10–12),^38,39^ which provide access to thioether-based macrocycles (peptides 14–17). Ultimately, considerations of synthetic accessibility and modularity guided the selection of cyclization strategies explored in this study.

Hence, particular emphasis was placed on lactam cyclization, which was selected as a synthetically reliable and conformationally well-defined approach, while systematically probing the effects of macrocycle size and topology (Table 1). Specifically, the first subset of analogues was designed to undergo ‘*head-to-side-chain*’ cyclization *via* lactam bridge formation between the C-terminal α-carboxyl group and the side-chain amine of diamino acids such as 2,4-diaminobutyric acid (Dab) (peptides 6 and 7), affording 20-membered macrocycles, or 2,3-diaminopropionic acid (Dap) (peptides 10 and 11), yielding 19-membered macrocycles. In parallel, additional analogues were designed to feature ‘*head-to-tail*’ cyclization between the C-terminal α-carboxyl group and the amino group of γ-aminobutyric acid (GABA) (peptides 8 and 9) or β-alanine (peptides 12 and 13). This strategy preserved the same 20- or 19-atom ring size, respectively, while altering the cyclization topology. Thioether-based analogues were generated through ‘*side-chain-to-tail*’ cyclization between a cysteine residue and an alkyl spacer in the N-terminus comprising either three or two methylene units, resulting in macrocycles of 20-membered (peptides 14 and 15) or 19-membered (peptides 16 and 17) ring size. Finally, to further probe functional outcomes, dPhe and dNal(2) residues were selectively incorporated at specific positions to recapitulate key pharmacophoric elements previously associated with agonist and antagonist reference sequences.

**Table 1:** Sequences and cyclization features of the cyclic melanocortin peptide analogues investigated in this study (peptides 6–17)^b^

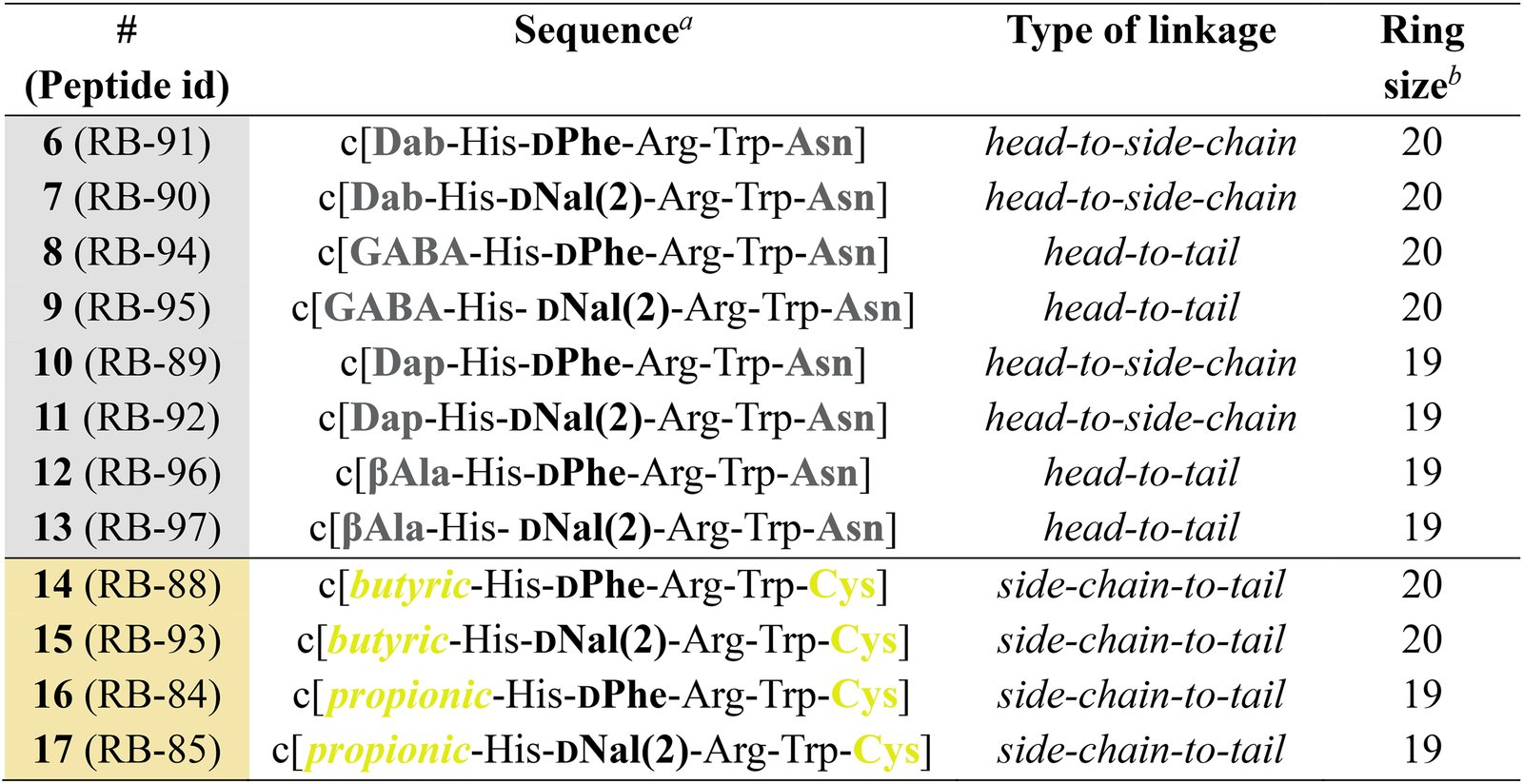

a Grey residues indicate amino acids involved in lactam bridge formation, whereas dark yellow residues indicate those involved in thioether cyclization. Bold residues [dPhe and dNal(2)] represent agonist- and antagonist-related pharmacophoric elements in the reference sequences (*e.g.*, **MT-II** and **SHU-9119**, respectively).

b Ring size is defined as the number of peptide backbone atoms involved in the cycle.

### Peptide synthesis

Peptides 6–17 were manually synthesized using ultrasound-assisted solid-phase peptide synthesis (SPPS), following 9-fluorenylmethoxycarbonyl (Fmoc) chemistry for the assembly of linear sequences (Scheme 1).^42^

**Scheme 1:**
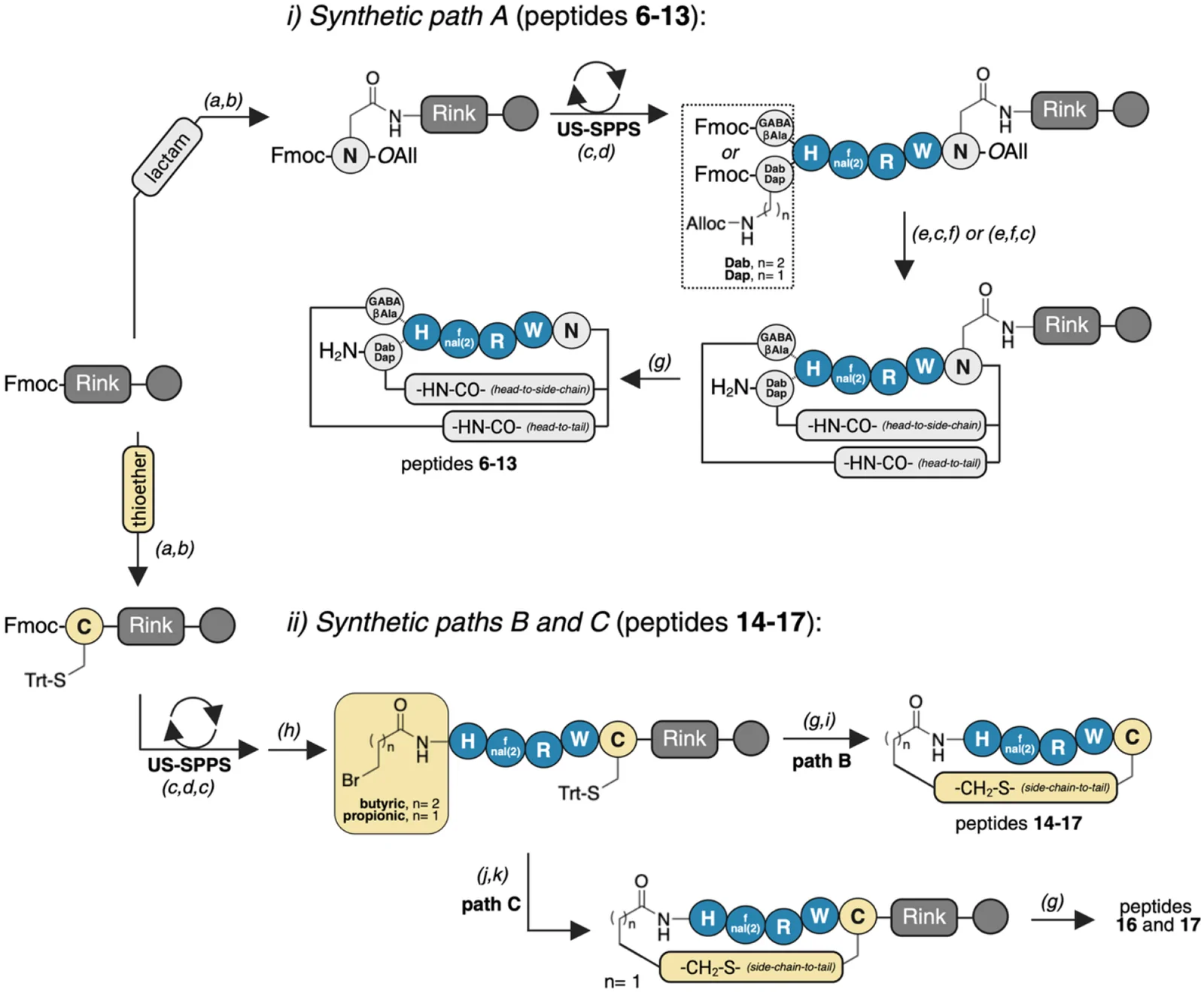
(a) 20% piperidine in DMF, 5 + 25 min; (b) Fmoc-AA, HBTU, HOBt (3 equiv. each), DIPEA (6 equiv.), in DMF, 2 h; (c) US-SPPS Fmoc-deprotection: 20% piperidine in DMF, 0.5 + 1 min; (d) US-SPPS coupling: Fmoc-AA (3 equiv.), HBTU/HOBt (3 equiv each.), DIPEA (6 equiv.) in DMF, 15 min; path A (for peptides 6–13): (e) Pd(PPh_3_)_4_ (0.15 equiv.), NDMBA (3 equiv.), DCM/DMF dry (3 : 2, v/v), 1 h × 2; (f) PyAOP (3 equiv.), DIPEA (6 equiv.) in DMF, 12 h; (g) TFA/H_2_O/TIS (95 : 2.5 : 2.5, v/v/v), 3 h. (h) 4-Bromobutyric or 3-bromopropionic acid acid (2 equiv.), DIC (1 equiv.) in dry THF, 12 h. Path B (for peptide 14–17): (i): DMAP (0.3 equiv.), DIPEA (3 equiv.) in H_2_O/MeCN (1 : 1, v/v), 12 h. Path C (for peptides 16 and 17): (j) Ag(CF_3_CO_2_) (0.4 equiv.) in DCM 2 × 15 min; (k) Cs_2_CO_3_ (4 equiv.) in dry DMF, 12 h.

After completion of the linear sequences, lactam-bridged analogues (peptides 6–13) were subjected to selective deprotection steps to enable intramolecular cyclization (Scheme 1, path A).^43,44^ Specifically, the allyl ester protecting group was removed from the α-carboxyl function of the residue originally introduced as a side-chain-anchored Asp-*O*All on the resin, together with the cleavage of the allyloxycarbonyl (Alloc) protecting group present in peptides 6, 7, 10 and 11, as previously reported.^43,45^ Following selective deprotection, intramolecular lactam formation was performed on the resin using (7-azabenzotriazol-1-yloxy)tripyrrolidinophosphonium hexafluorophosphate (PyAOP) in the presence of 1-hydroxy-7-azabenzotriazole (HOAt) as an additive and *N*,*N*-diisopropylethylamine (DIEA) as base.^43,45^ Subsequently, the N^α^-terminal Fmoc-protecting group was removed. This cyclization strategy afforded lactam-based ‘*head-to-side-chain*’ macrocycles. For peptides 8, 9, 12 and 13, lactam cyclization was performed after Fmoc removal, resulting exclusively in ‘*head-to-tail*’ macrocyclization. Upon completion of cyclization, peptides were cleaved from the solid support, affording the corresponding crude cyclic products.

For thioether-based analogues (peptides 14–17), linear peptide sequences were first assembled on resin, after which 4-bromobutyric acid (peptides 14 and 15) or 3-bromopropionic acid (peptides 16 and 17) was coupled at the N-terminus. This coupling step was performed under modified conditions relative to standard amino acid coupling. *N*,*N*′-diisopropylcarbodiimide (DIC) and 1-hydroxybenzotriazole (HOBt) were employed for 2 h at room temperature.^46,47^ Following cleavage from the resin using a trifluoroacetic acid (TFA)-based cocktail, intramolecular thioether macrocyclization was accomplished in solution *via* nucleophilic substitution between the cysteine thiol and the N-terminal bromoalkyl group, yielding the crude cyclic peptides (Scheme 1, path B).^48^ Alternatively, peptides 16 and 17 were synthesized through our recently developed Ag(CF_3_CO_2_)-mediated on-resin *S*-detritylation strategy, enabling selective cysteine deprotection under SPPS conditions followed by intramolecular cyclization, to afford the corresponding on-resin thioethers, followed by cleavage to recovery crude cyclic peptides (Scheme 1, path C).^49^

Crude peptides (6–17) were purified by reversed-phase high-performance liquid chromatography (RP-HPLC). Final products were obtained with purities greater than 97%, as confirmed by analytical RP-HPLC, and their molecular masses were verified by electrospray ionization mass spectrometry (ESI-MS).

### Conformational study through CD analysis

The influence of the type of macrocyclization on the solution conformation was investigated using circular dichroism (CD) spectroscopy (Fig. 3). The 20-membered lactam analogues (peptides 6–9, Fig. 3A) predominantly adopted a disordered conformation, except for peptide 6, which displayed a β-type conformation characterized by a minimum at 210 nm. Interestingly, peptide 7 showed an unusual minimum at around 235 nm, likely arising from intramolecular aromatic clusters involving the dNal(2), His, and Trp side chains, stabilized by π–π stacking interactions, as observed elsewhere.^50^ Similarly, the 19-membered lactam series (peptides 10–13, Fig. 3B) adopted a disordered conformation, with a minimum around 200 nm. Within this series, peptide 13 also exhibited the unusual negative band at 235 nm, resembling that observed for peptide 7 and potentially arising from π–π stacking interactions among the aromatic side chains of dNal(2), His, and Trp.

**Fig. 3:**
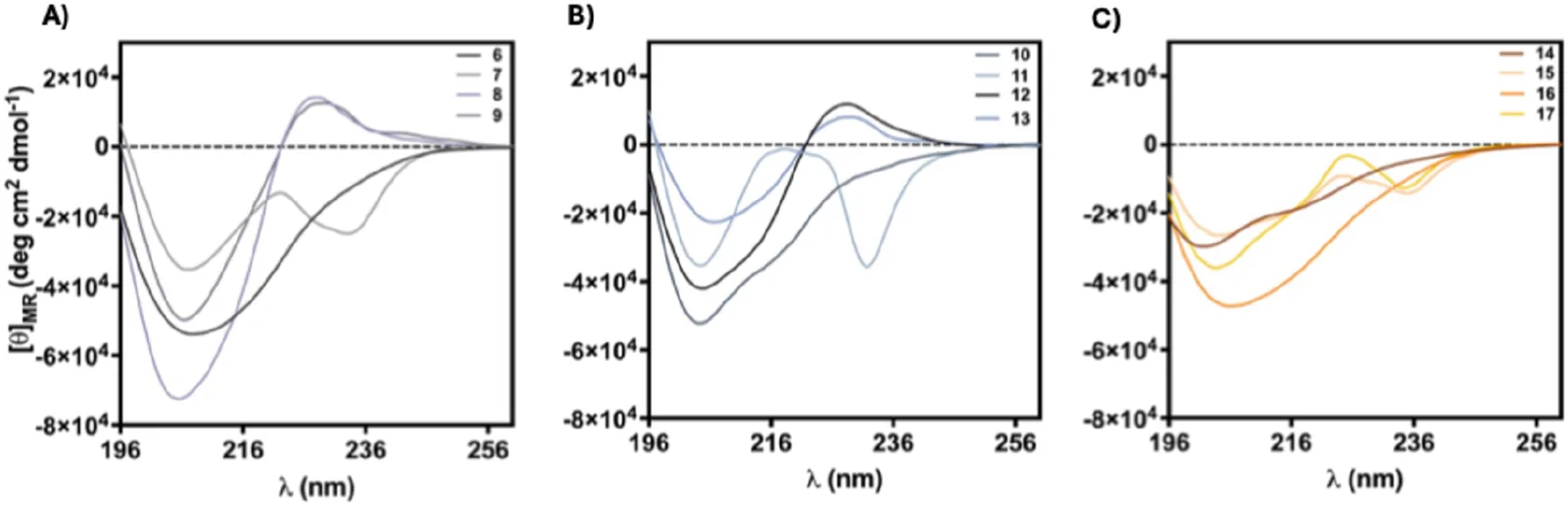
CD spectra of 20- and 19-membered lactam (6–13, panels A and B) and thioether (14–17, panel C) peptide series in solution.

Regarding the 20- and 19-membered thioether analogues (Fig. 3C), peptides 14–17 predominantly adopted disordered conformations, characterized by a minimum around 200 nm, except for peptide 16, which showed a β-type conformation. As observed for lactam analogues 7 and 13, peptides 15 and 17 exhibited an unusual minimum at 235 nm. However, this characteristic band was observed exclusively in dNal(2)-containing analogues combined with specific cyclization topologies, particularly the ‘*head-to-side-chain*’ topology, whereas among the ‘*head-to-tail*’ analogues it was observed only for peptide 13. These findings suggested that the combination of dNal(2) with specific macrocyclic constraints may increase the local conformational rigidity, thereby facilitating the formation of an intramolecular aromatic cluster stabilized by π–π stacking interactions.

### Concentration–response profile for cAMP accumulation

All synthesized peptides were evaluated at *h*MC3R- and *h*MC4R-expressing HEK-293 cell lines to determine agonist activities. We routinely verify receptor identity in these cell lines by quantitative reverse-transcriptase PCR to ensure the correct receptor subtype is assigned to each cell line during maintenance and experimentation. Receptor expression levels were measured by radioligand saturation binding experiments using [^125^I]-NDP-MSH for each cell line. *B*_max_ values ranged from 2.2 pmol mg^−1^ of total homogenate protein to 455 fmol mg^−1^ of total homogenate protein for *h*MC3R and *h*MC4R, respectively. Agonist activities were expressed as pEC_50_, *E*_max_, and relative (to α-MSH) transduction ratio [Δlog(*E*_max_/EC_50_)] to account for the fivefold difference in receptor expression and allow direct comparison of agonist activities, which reflect *G*_s_-mediated cAMP accumulation (Table 2, Fig. 4). Specifically, *E*_max_ values were normalized to the maximal response elicited by α-MSH, which was defined as 100%. In addition, IC_50_ functional inhibition potency values were reported only for compounds showing clear sigmoidal inhibition greater than 50% of α-MSH-induced responses at an EC_90_-equivalent concentration. These values are presented in Table 3 as a measure of functional antagonism. Compounds were designed to systematically explore the impact of cyclization strategy, ring size, and bridge chemistry on melanocortin receptor activity.

**Table 2:** Characterization of the concentration–response profile for cAMP accumulation by *h*MC3R and *h*MC4R for the indicated compounds in the agonist mode

Peptide id (#)	MC3R			MC4R		
	pEC_50_ (EC_50_)^a^	Efficacy^b^ (%)	Relative transduction ratio^d^	pEC_50_ (EC_50_)^a^	Efficacy^b^ (%)	Relative transduction ratio^d^
**MT-II** c	7.77 ± 0.10	100		8.00 ± 0.01	100	
**RB-91** (6)	6.62 (2.41 × 10^−0.7^)	85.5	−2.226	8.37 (4.23 × 10^−0.9^)	98.0	−0.080
**RB-90** (7)	6.33 (4.70 × 10^−0.7^)	4.9	−3.716	6.86 (1.38 × 10^−0.7^)	14.6	−2.415
**RB-94** (8)	6.58 (2.61 × 10^−0.7^)	93.1	−2.223	7.73 (1.85 × 10^−0.8^)	102.3	−0.702
**RB-95** (9)	6.77 (1.68 × 10^−0.7^)	5.8	−3.210	7.20 (6.31 × 10^−0.8^)	21.2	−1.907
**RB-89** (10)	6.88 (1.33 × 10^−0.7^)	97.4	−1.910	8.07 (8.45 × 10^−0.9^)	105.6	−0.350
**RB-92** (11)	6.87 (1.36 × 10^−0.7^)	11.5	−2.837	6.96 (1.08 × 10^−0.7^)	57.6	−1.715
**RB-96** (12)	5.62 (2.42 × 10^−0.6^)	101.9	−3.152	6.94 (1.15 × 10^−0.7^)	96.9	−1.520
**RB-97** (13)	6.83 (1.48 × 10^−0.7^)	86.2	−2.013	7.80 (1.57 × 10^−0.8^)	93.1	−0.674
**RB-88** (14)	8.02 (9.64 × 10^−0.9^)	95.3	−0.783	9.55 (2.80 × 10^−10^)	109.3	1.142
**RB-93** (15)	7.72 (1.89 × 10^−0.8^)	8.6	−2.100	8.48 (3.31 × 10^−0.9^)	21.4	−0.624
**RB-84** (16)	6.68 (2.07 × 10^−0.7^)	83.7	−2.168	7.69 (2.03 × 10^−0.8^)	97.3	−0.765
**RB-85** (17)	6.57 (2.71 × 10^−0.7^)	9.2	−3.222	7.21 (6.22 × 10^−0.8^)	16.1	−2.030

a pEC_50_ values were calculated as the negative logarithm (base 10) of EC_50_ values expressed in molar units. pEC_50_ = −log(EC_50_[*M*]).

b Top plateau = *E*_max_. *E*_max_ values were normalized to the maximal response elicited by α-MSH, defined as 100%.

c Data taken from ref. 24.

d Relative to intraplate α-MSH transduction ratio [Δ_α-MSH_log(*E*_max_/EC_50_)] values. Negative values indicate less agonist activity relative to α-MSH. Positive values indicate greater agonist activity relative to α-MSH.

**Fig. 4:**
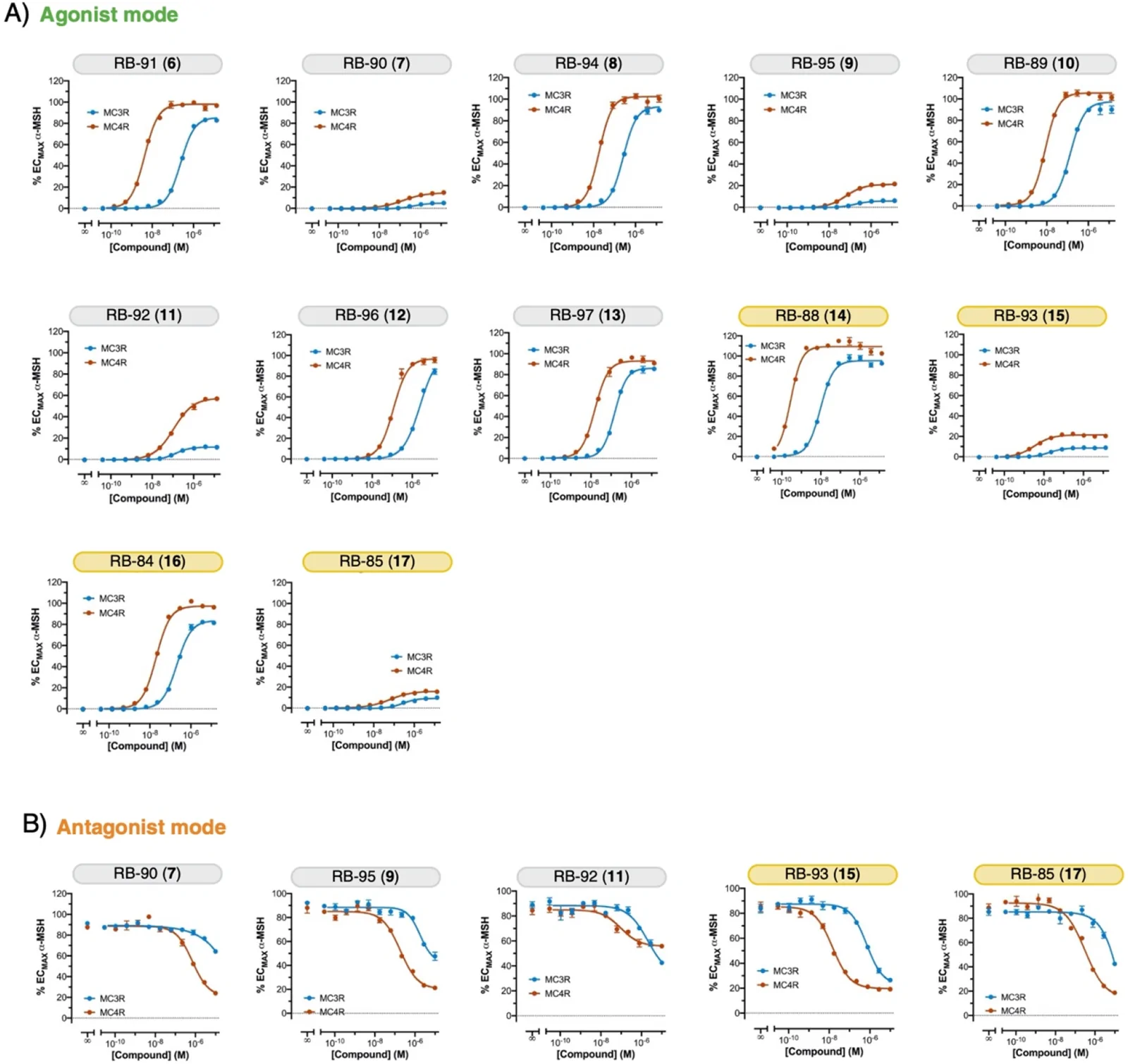
Concentration–response curves of the cyclic peptide analogues at *h*MC3R (shown in blue) and *h*MC4R (shown in red). (A) Agonist activity evaluated by cAMP accumulation following treatment with peptides 6–17. (B) Antagonist activity of selected analogues evaluated as inhibition of α-MSH-induced cAMP accumulation. Data points represent the mean ± standard error from an experiment performed in triplicate.

**Table 3:** Characterization of the inhibition of α-MSH-induced cAMP accumulation at *h*MC3R and *h*MC4R for selected compounds

Peptide id (#)	*h*MC3R		*h*MC4R	
	pIC_50_ (IC_50_)^a^	Inhibition^b^ (%)	pIC_50_ (IC_50_)^a^	Inhibition^b^ (%)
**SHU-9119** c	7.08 ± 0.31	*n.a.*	7.42 ± 0.20	*n.a.*
**RB-90** (7)	*n.d.*	0	6.15 (7.06 × 10^−0.7^)	68
**RB-95** (9)	5.74 (1.81 × 10^−0.6^)	46	6.73 (1.85 × 10^−0.7^)	65
**RB-92** (11)	5.68 (2.07 × 10^−0.6^)	57	7.02 (9.66 × 10^−0.8^)	29
**RB-93** (15)	6.16 (6.97 × 10^−0.7^)	65	7.83 (1.49 × 10^−0.8^)	66
**RB-85** (17)	4.45 (3.58 × 10^−0.5^)		6.50 (3.16 × 10^−0.7^)	78

a IC_50_ values represent inhibition of α-MSH-induced responses. Values are reported only for compounds showing a clear sigmoidal inhibition curve. *n.d.* = not determined due to lack of a reliable inhibition curve.

b Inhibition values correspond to the fitted curve span, calculated as the difference between the upper and lower plateaus of the concentration–response curve.

c pIC_50_ data referred to **SHU-9119** are reported in ref. 24. *n.a.* = not available.

Focusing on the 20-membered lactam series (peptides 6–9), peptides 6 (**RB-91**) and 7 (**RB-90**) provided initial insight into how the pharmacophoric residue modulates functional outcomes within a conserved macrocyclic scaffold. Peptide 6, containing a dPhe residue, behaved as an agonist at both receptors, showing higher functional potency at *h*MC4R [pEC_50_ = 8.37, *E*_max_ ≈ 98%, Δlog(*E*_max_/EC_50_) = −0.08] than at *h*MC3R [pEC_50_ = 6.62, *E*_max_ ≈ 85%, Δlog(*E*_max_/EC_50_) = −2.23]. This profile indicates a preference for *h*MC4R, primarily due to differences in functional potency that yield a relative transduction ratio to α-MSH closer to zero, consistent with agonist activity similar to that of α-MSH at *h*MC4R. In contrast, the dNal(2)-containing analogue peptide 7 showed negligible agonist activity at both receptors (*E*_max_ ≈ 5% at *h*MC3R and ≈ 15% at *h*MC4R). However, in antagonist mode, peptide 7 inhibited the α-MSH-induced response at *h*MC4R (pIC_50_ = 6.15), whereas no reliable inhibition was observed at *h*MC3R, indicating a preferential functional antagonist profile at *h*MC4R. A similar trend was observed for peptides 8 (**RB-94**) and 9 (**RB-95**). Peptide 8 (dPhe-containing) acted as a highly efficacious agonist at *h*MC4R [pEC_50_ = 7.73, *E*_max_ ≈ 102%, Δlog(*E*_max_/EC_50_) = −0.70] and as a less potent, though still highly efficacious, agonist at *h*MC3R [pEC_50_ = 6.58, *E*_max_ ≈ 93%, Δlog(*E*_max_/EC_50_) = −2.22], confirming a preference for *h*MC4R. Conversely, peptide 9, dNal(2)-containing, showed minimal agonist activity (*E*_max_ ≈ 6% at *h*MC3R and ≈ 21% at *h*MC4R) and behaved predominantly as a functional antagonist, inhibiting α-MSH responses at both receptors with higher potency at *h*MC4R (pIC_50_ = 6.73 *vs.* 5.74 at *h*MC3R).

Reducing the macrocycle size to 19 members (peptides 10–13) further modulated potency and efficacy. Peptide 10 (**RB-89**), dPhe-containing, displayed a potent agonist profile at both receptors, acting as a highly efficacious agonist at *h*MC4R [pEC_50_ = 8.07, *E*_max_ ≈ 105%, Δlog(*E*_max_/EC_50_) = −0.35] and at *h*MC3R [pEC_50_ = 6.88, *E*_max_ ≈ 97%, Δlog(*E*_max_/EC_50_) = −1.91], with a clear preference for *h*MC4R driven by higher potency. Replacing dPhe with dNal(2) in peptide 11 (**RB-92**) markedly reduced efficacy, yielding partial agonist activity at *h*MC4R (*E*_max_ ≈ 57%) and minimal activation at *h*MC3R (*E*_max_ ≈ 11%). In antagonist mode, peptide 11 inhibited α-MSH-induced responses at both receptors, again showing higher potency at *h*MC4R (pIC_50_ = 7.02 *vs.* 5.68), thus displaying a mixed partial agonist/functional antagonist profile. Peptide 12 (**RB-96**), a dPhe-containing ‘*head-to-tail*’ analogue, retained high efficacy at both receptors (*E*_max_ ≈ 97% at *h*MC4R and ≈ 102% at *h*MC3R) but showed reduced potency and relative transduction ratios compared to peptide 10, particularly at *h*MC3R [pEC_50_ = 5.62 *vs.* 6.88, Δlog(*E*_max_/EC_50_) = −3.15 *vs.* −1.91], while maintaining preference for *h*MC4R (pEC_50_ = 6.94, Δlog(*E*_max_/EC_50_) = −1.52). This indicated that, within the dPhe-containing 19-membered series, cyclization topology has a stronger impact on potency than on maximal efficacy. Peptide 13 (**RB-97**), the corresponding dNal(2) analogue, displayed an agonist profile at both receptors (pEC_50_= 7.80 at *h*MC4R and 6.83 at *h*MC3R), with substantial efficacy (*E*_max_ ≈ 93% and 86%, respectively). In contrast to peptide 11, no reliable antagonist activity was observed. Notably, despite containing a dNal(2) residue, peptide 13 behaved as an agonist, in contrast to the corresponding ‘*head-to-side-chain*’ analogue 11, which showed a predominantly functional antagonist profile.

A second subset of analogues incorporating a thioether bridge was evaluated to explore the functional profiles associated with an alternative macrocyclic chemistry (peptides 14–17). Peptide 14 (**RB-88**), a thioether-bridged analogue containing a dPhe residue, acted as an agonist, with subnanomolar activity at *h*MC4R [pEC_50_ = 9.55, *E*_max_ ≈ 109%, Δlog(*E*_max_/EC_50_) = 1.14] and high potency at *h*MC3R [pEC_50_ = 8.02, *E*_max_ ≈ 95%, Δlog(*E*_max_/EC_50_) = −0.78], confirming a strong preference for *h*MC4R. Notably, the remarkable activity of peptide 14 indicates that its thioether-containing 20-membered architecture provides a particularly favourable structural context for *h*MC4R activation. In contrast, the corresponding dNal(2)-containing analogue 15 (**RB-93**) showed weak partial agonist activity (*E*_max_ ≈ 21% at *h*MC4R and ≈ 9% at *h*MC3R) and inhibited α-MSH-induced responses at both receptors, thus displaying a weak partial agonist/functional antagonist profile, with higher apparent inhibitory potency at *h*MC4R (pIC_50_ = 7.83 *vs.* 6.16 at *h*MC3R).

Peptides 16 (**RB-84**) and 17 (**RB-85**), featuring 19-membered rings, displayed reduced potency relative to their 20-membered counterparts, indicating that ring size affects receptor activation. In particular, peptide 16 showed good agonist activity at *h*MC4R [pEC_50_ ≈ 7.7, *E*_max_ ≈ 95–100%, Δlog(*E*_max_/EC_50_) = −0.77], but with a more limited preference for *h*MC4R over *h*MC3R [ΔΔlog(*E*_max_/EC_50_) = 1.4, 25-fold selectivity]. The corresponding dNal(2)-containing analogue 17 also exhibited negligible agonist activity, with *E*_max_ values of approximately 9% at *h*MC3R and 16% at *h*MC4R, indicating a reduction in efficacy associated with dPhe substitution with dNal(2). However, peptide 17 displayed a functional antagonist profile at both receptors with a clear preference for *h*MC4R (pIC_50_ = 6.50) over *h*MC3R (pIC_50_ = 4.45). Although its antagonist potency was lower than that of the 20-membered analogue peptide 15, the replacement of dPhe with dNal(2) retained an *h*MC4R-preferential functional antagonist profile.

### Structure–activity relationship analysis

The SAR from this series of cyclic melanocortin peptide analogues highlighted the combined influence of macrocycle size, cyclization topology, and the dPhe/dNal(2) pharmacophoric substitution on potency, efficacy and functional preference between *h*MC4R and *h*MC3R (Fig. 5 and 6). Lactam and thioether bridges were explored as macrocyclization strategies to generate downsized 20- and 19-membered analogues and to determine how these structural modifications affected receptor response profiles. Across the series, the dPhe/dNal(2) substitution emerged as a major determinant of the agonist/antagonist balance, whose functional impact was strongly modulated by the overall macrocyclic architecture, including ring size and cyclization topology. All peptides exhibited a functional preference for *h*MC4R, as shown by fold-selectivity values greater than unity (Fig. 6).

**Fig. 5:**
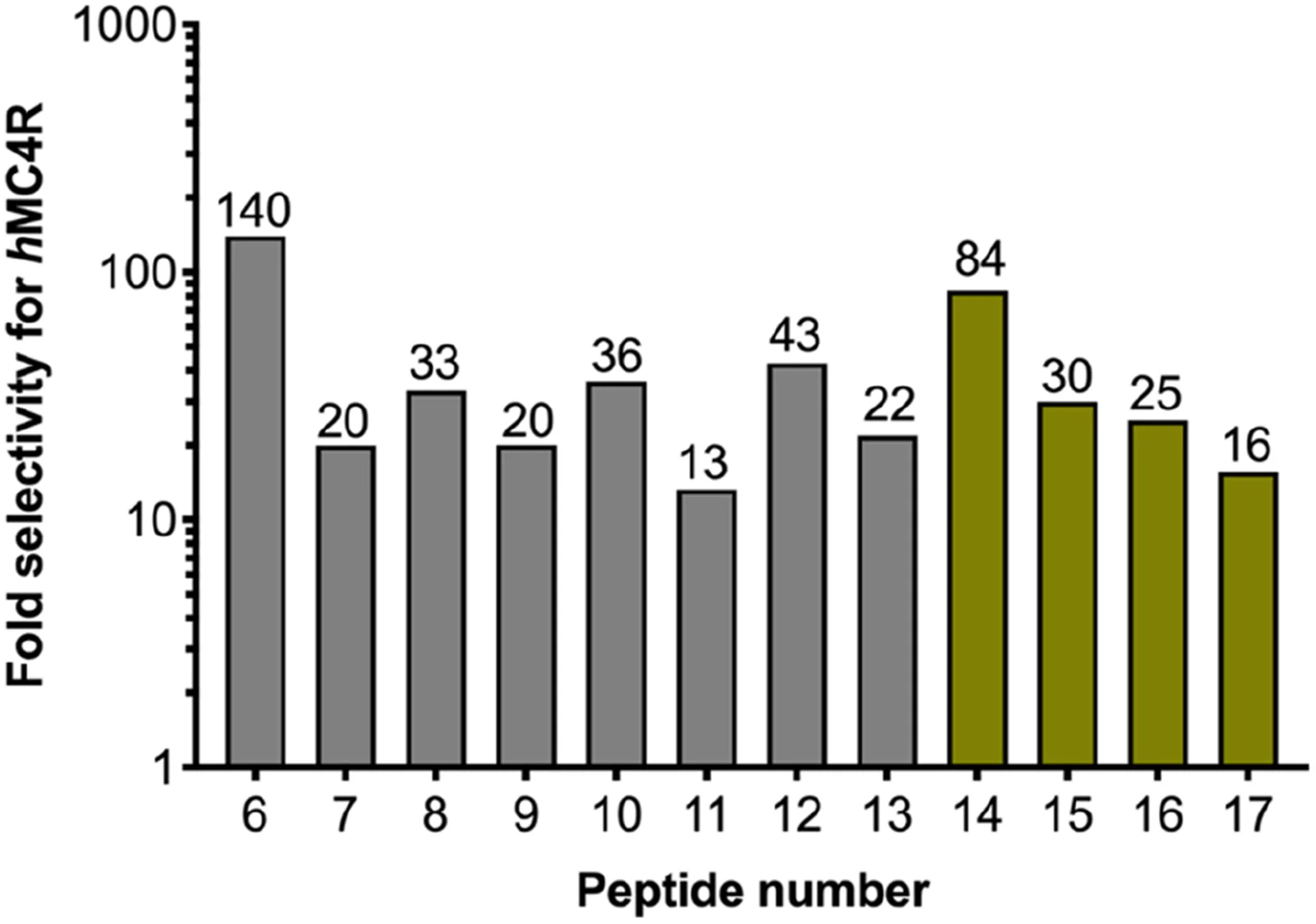
Agonist functional selectivity of cyclic melanocortin peptide analogues 6–17 between *h*MC3R and *h*MC4R. Lactam-bridged analogues are shown as grey bars, whereas thioether-bridged analogues are shown as yellow bars. Selectivity ratios were obtained by calculating the antilog of ΔΔlog(*E*_max_/EC_50_) values for *h*MC4R and *h*MC3R, respectively, as described in ref. 52.

**Fig. 6:**
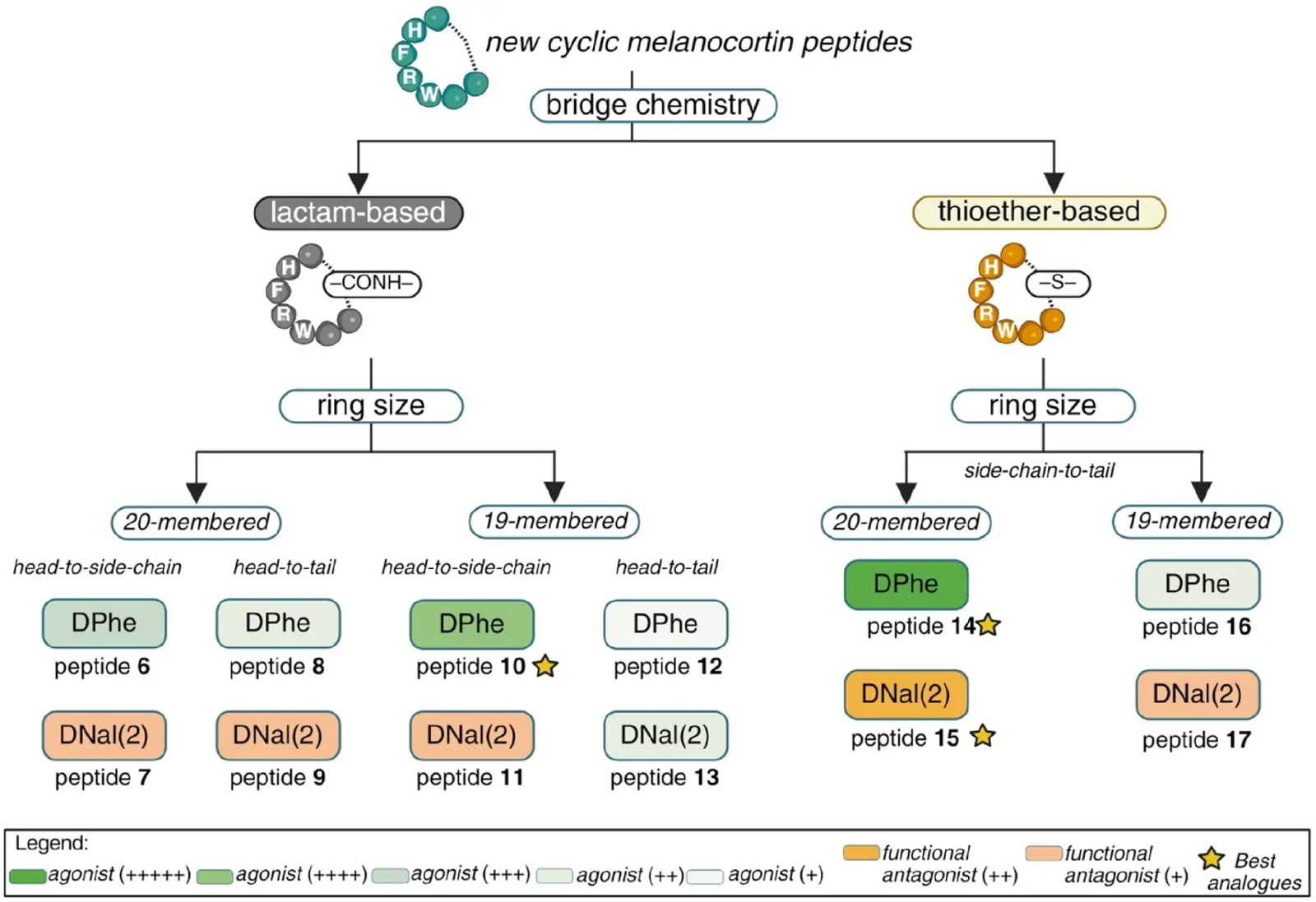
SAR overview of the cyclic melanocortin peptide library. Analogues are grouped according to bridge chemistry, ring size, cyclization topology and aromatic residue [dPhe or dNal(2)]. Colour shading reflects the relative agonist or antagonist potency of the analogues, while stars identify the best-performing analogues.

Within the 20-membered lactam scaffold, retention of the dPhe residue promoted agonist activity, as observed for peptides 6 and 8. Both analogues behaved as agonists at *h*MC3R and *h*MC4R, with a preferential response toward *h*MC4R. This preference was mainly associated with higher potency at *h*MC4R, as reflected by the pEC_50_ values of peptides 6 and 8 at *h*MC4R relative to *h*MC3R. Both peptides also showed greater efficacy at *h*MC4R (*E*_max_ 98% and 102%, respectively). Moreover, peptide 6 exhibited the strongest functional preference for *h*MC4R among the lactam series, with a ΔΔlog(*E*_max_/EC_50_) value of 2.15, corresponding to 140-fold selectivity for *h*MC4R over *h*MC3R. Comparison between peptides 6 and 8 revealed that changing the cyclization topology from ‘*head-to-side-chain*’ to ‘*head-to-tail*’, and the concomitant absence of the additional positive charge at the N-terminus, did not alter the agonist profile of the compounds. Thus, in the 20-membered lactam series, the presence of dPhe was consistently associated with agonist activity, whereas cyclization topology further influenced potency and receptor preference. Conversely, replacement of dPhe with dNal(2) in the 20-membered lactam subset shifted the activity profile toward reduced agonist efficacy and functional antagonism, as observed for peptides 7 and 9, supporting an important contribution of the dPhe/dNal(2) substitution to the functional activity within this macrocyclic scaffold.^51^ Within this subset, the ‘*head-to-tail*’ analogue (peptide 9) showed a more pronounced functional antagonist profile than the corresponding ‘*head-to-side-chain*’ analogue (peptide 7). Moreover, the replacement of dPhe with dNal(2) impacted selectivity, reducing *h*MC4R preference, as reflected by the selectivity ratio of 20 [based on a ΔΔlog(*E*_max_/EC_50_) value of 1.3] observed for peptide 9. These results indicate that dPhe/dNal(2) substitution strongly influences the functional profile within the 20-membered lactam subset, although its effect on agonism/antagonism and *h*MC4R preference remains dependent on the surrounding macrocyclic architecture.

In the 19-membered lactam subset, analogues 10 and 12 with dPhe retained agonist activity at both receptors. However, switching to ‘*head-to-tail*’ cyclization topology (peptide 12) reduced potency and the corresponding relative transduction ratio, particularly at *h*MC3R [pEC_50_ = 5.62 *vs.* 6.88, Δlog(*E*_max_/EC_50_) = −3.15 *vs.* −1.91]. The dNal(2)-containing ‘*head-to-side-chain*’ analogue (peptide 11) displayed a mixed functional profile, combining partial agonism and inhibition of α-MSH-induced responses, with a markedly reduced *h*MC4R preference (selectivity ratio of 13, the lowest value in the series). In contrast, the corresponding ‘*head-to-tail*’ analogue (peptide 13) retained substantial agonist efficacy at both receptors (*E*_max_ ≈ 93% and 86%, respectively) and restored a measurable preference for *h*MC4R (22-fold selectivity), despite retaining the dNal(2) residue. This result suggests that, in the 19-membered lactam scaffold, the functional effect associated with dNal(2) is strongly dependent on cyclization topology. The thioether-containing subset further illustrated how alternative macrocyclic architectures can modulate the functional profile of these analogues. Within this subset, the 20-membered dPhe-containing analogue (peptide 14) displayed a potent agonist profile (*E*_max_ ≈ 109% and 95%), with one of the highest *h*MC4R preferences of the series (84-fold). This result indicated that the combination of a thioether linkage, a 20-membered macrocycle, and the dPhe residue provides a favourable agonist profile and pronounced *h*MC4R activation and discrimination. Ring contraction to the corresponding 19-membered analogue (peptide 16) decreased receptor preference to 25-fold. This comparison indicates that macrocycle size influences receptor preference within the thioether-bridged subset. Replacement of dPhe with dNal(2) shifted the functional profiles of the 20- and 19-membered thioether analogues 15 and 17 toward reduced intrinsic agonist efficacy and inhibition of α-MSH-induced responses. However, functional antagonism was more pronounced for the 20-membered analogue (peptide 15), indicating that, also within the thioether series, macrocycle size contributed to fine-tuning antagonist potency and receptor preference. Although peptide 15 retained a six-fold preference for *h*MC4R over *h*MC3R, its receptor preference was markedly lower than that of the corresponding dPhe analogue 14 (34-fold). Further ring contraction to peptide 17 reduced *h*MC4R preference to four-fold. Nevertheless, the thioether-bridged analogues generally showed stronger *h*MC4R preference than the corresponding dNal(2)-containing lactam analogues examined in this study, supporting bridge chemistry as a major determinant of receptor preference. These results demonstrate that the functional profiles of the cyclic analogues arise from the interplay between pharmacophore identity and the overall macrocyclic architecture, including bridge chemistry, macrocycle size, and cyclization topology. The dPhe/dNal(2) substitution emerged as a major determinant of the agonist/antagonist balance. However, the final functional activity remained strongly dependent on the macrocyclic architecture, as particularly illustrated by the distinct profiles of the dNal(2)-containing peptides 11 and 13. The highest *h*MC4R preference was observed for the 20-membered dPhe-containing analogues, particularly peptide 6 (140-fold) and peptide 14 (84-fold), whereas incorporation of dNal(2) was generally associated with reduced receptor discrimination. Nevertheless, the extent of this reduction depended strongly on macrocycle architecture.

Among the analogues showing inhibition of α-MSH-induced responses, peptides 9, 11 and 15 displayed the strongest preference for *h*MC4R, showing *h*MC4R/*h*MC3R selectivity ratios ≥10-fold (see Fig. 5 and Table S2). Notably, the preferential functional profile toward *h*MC4R observed for the present His-containing analogues is consistent with that previously reported for the corresponding Pro-containing lactam series.^30^ Despite restoring the canonical His residue, the present 19- and 20-membered lactam macrocycles retained a clear preference for *h*MC4R over *h*MC3R. Collectively, these findings suggest that, within this class of downsized lactam macrocycles, receptor preference is largely governed by the overall macrocyclic architecture rather than by replacement of the conserved His residue with Pro.

To provide a structural framework for the observed SAR, peptide 6 was selected as a representative analogue for conformational prediction, since its CD spectrum was the most consistent with a defined β-type conformation within the present series. Conformational sampling identified a recurrent low-energy family characterized by a type II-like β-turn involving the Dab^1^–His^2^–dPhe^3^–Arg^4^ region. In the representative conformer (Fig. S14, SI), the C_α_(Dab^1^)–C_α_(Arg^4^) distance was approximately 4.6 Å, consistent with the commonly used geometric criterion for β-turn assignment (<7 Å).^53^ The corresponding backbone dihedral angles [His^2^: *φ* = −71.1°, *ψ* = +77.6°; dPhe^3^: *φ* = +105.2°, *ψ* = +6.6°] further supported a type II-like geometry. This predicted conformational preference is consistent with the established propensity of cyclic melanocortin ligands to organize their pharmacophoric region into turn-like structures. Previous NMR studies of **MT-II** and **SHU-9119** identified β-turn motifs involving the central pharmacophoric region and revealed differences in the conformational behaviour of the dPhe and dNal(2) side chains.^53^ More recently, structural studies based on the **SHU-9119**/*h*MC4R complex have further highlighted that the macrocyclic constraint can influence ligand geometry and the spatial presentation of pharmacophoric residues.^24^ In particular, modifications of the cyclization motif were proposed to affect the macrocyclic backbone conformation and consequently ligand positioning, even though the cyclization tether itself does not directly interact with the receptor. Within this framework, macrocycle contraction and changes in cyclization topology may influence the functional activity of the present analogues by altering the conformational preferences of the scaffold and, consequently, the spatial presentation of the conserved His–dPhe/dNal(2)–Arg–Trp pharmacophore. The type II-like β-turn predicted for peptide 6 provides a plausible structural correlate for the β-type conformational signature observed by CD and may contribute to the favourable agonist profile of this analogue. In this context, the agonist profile retained by the dNal(2)-containing peptide 13 suggests that the functional consequence of the dPhe/dNal(2) substitution strongly depends on the conformational constraints imposed by cyclization topology. Thus, the observed agonist/functional antagonist balance may arise from the interplay between pharmacophore identity and macrocyclic architecture rather than from either feature independently.

### Human serum stability

The proteolytic stability of selected peptide analogues 6, 10, 14 and 15 was evaluated in human serum. These compounds were selected to combine pharmacological relevance with representative structural diversity within the series. In particular, lactam analogues 6 and 10 allowed comparison of 20- and 19-membered ‘*head-to-side-chain*’ macrocycles, whereas the 20-membered thioether analogues 14 and 15 enabled evaluations of the effect of the dPhe/dNal(2) substitution within the same macrocyclic architecture. Peptide 14 was also prioritized because of its particularly favourable *h*MC4R functional profile. For the serum stability assay, each peptide was incubated in 90% human serum at 37 ± 1 °C and analyzed over a 24 h period (Fig. 7). The percentage of intact peptide at different time points (0, 2, 4, 8 and 24 h) was determined by calculating the corresponding peak area from HPLC chromatograms, as elsewhere reported.^45^ The degradation profile of these analogues was compared with that of **MT-II**, and some of the selected compounds displayed improved serum stability compared with the reference compound, indicating that the applied structural modifications contribute to enhanced resistance to proteolytic degradation. Among the lactam-bridged analogues, 6 and 10, characterized by a ‘*head-to-side-chain*’ cyclization motif affording 20- and 19-membered macrocycles, respectively, showed a marked resistance to proteolytic degradation. Indeed, 6 and 10 retained 70% and 80% of the intact peptide after 4 h, respectively. Notably, peptide 10 showed a more favourable stability profile than 6, remaining approximately 75% intact after 8 h, and still retaining about 55% after 24 h, whereas peptide 6 decreased to ∼20% after 8 h. This behaviour is consistent with a possible contribution of the ‘*head-to-side-chain*’ lactam constraint to reduced proteolytic accessibility. However, the improved long-term stability of peptide 10 suggests that the smaller 19-membered macrocycle may contribute to reduced proteolytic accessibility. A different degradation behaviour was observed for the thioether-bridged analogues 14 and 15. These peptides are characterized by ‘*side-chain-to-tail*’ cyclization *via* the thioether motif, affording 20-membered macrocycles. Peptide 14 displayed high short-term stability, with ∼87% of the peptide remaining intact after 4 h. Its integrity then gradually decreased at later time points, reaching about 40% after 24 h. This profile indicates that the 20-membered thioether-containing architecture is compatible with substantial resistance to serum degradation. Peptide 15 differs from peptide 14 by the presence of dNal(2) instead of dPhe. Its stability was already reduced to 70% after 2 h, however, it appeared to be more stable over time, retaining about 60% of the intact peptide after 8 h and showing ∼50% stability after 24 h, similarly to **MT-II**. The different early degradation profile of 15 compared with 14 may reflect changes associated with the dPhe-to-dNal(2) substitution, potentially including differences in conformational behavior and accessibility of protease-sensitive regions. Interestingly, after the initial degradation phase, 15 showed only a moderate decrease over time. These data suggest that serum stability within the selected analogues reflects the interplay of multiple structural features, including cyclization topology, ring size and the nature of the pharmacophoric aromatic residue. Importantly, serum stability and functional potency should be considered as distinct optimization parameters, as illustrated by peptide 14, which combines very high *h*MC4R potency with moderate long-term serum stability, and peptide 10, which shows greater long-term stability despite lower *h*MC4R potency.

**Fig. 7:**
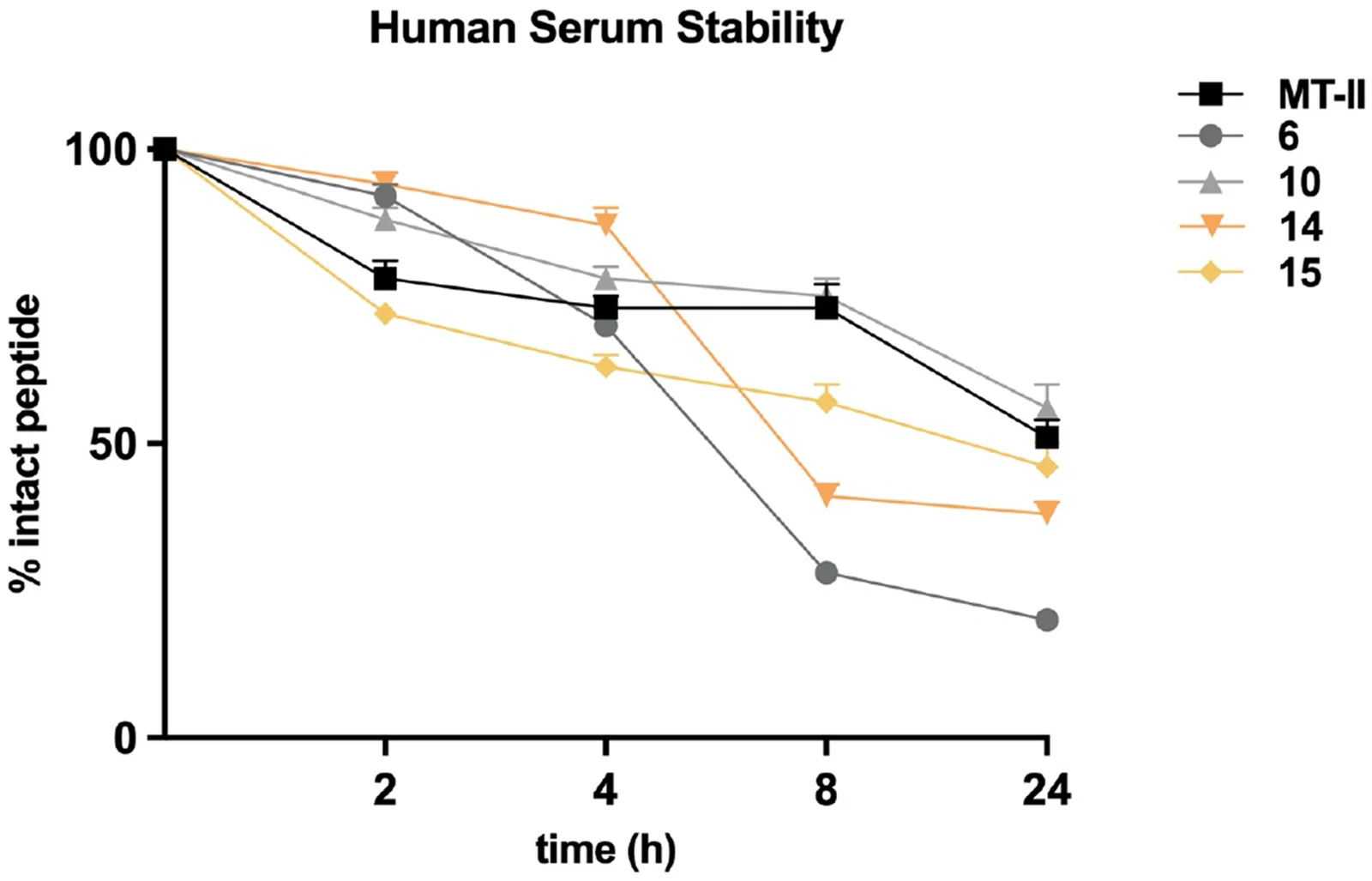
Proteolytic stability of **MT-II** lactam-bridged analogues 6 and 10, and thioether-bridged 14 and 15 in human serum at 37 ± 1 °C, over 24 h, at different time intervals. Data are expressed as the percentage of intact peptide at different time points. Error bars represent standard error.

## Conclusions

The development of these novel cyclic-constrained peptides represents a step forward toward achieving receptor-specific modulation of the melanocortin system. By elucidating the functional and structural properties of these analogues, we provide a foundation for future investigations into their potential in metabolic disorders, inflammation, and beyond, correlated with *h*MC3R and *h*MC4R. In particular, we have demonstrated that thioether bridges and dPhe pharmacophores enhance *h*MC4R preference; however, the mechanistic basis (whether conformational, electronic, or binding-mode-dependent) remains unclear. Some analogues (*e.g.*, 11 and 13) showed topology-dependent functional divergence that was not predicted by pharmacophore alone, indicating that additional structural determinants govern selectivity. Specifically, the 20- and 19-membered lactam-bridged analogues displayed agonist activity, while 20- and 19-membered thioether-bridged analogues proved to be more sensitive to macrocycle geometry. Among all compounds, peptide 14 emerged as the best analog, with sub-nanomolar agonist potency at *h*MC4R and high selectivity. In addition, the substitution of dPhe with dNal(2) within the His–dPhe/dNal(2)–Arg–Trp pharmacophore for thioether-bridged analogues shifted the functional profile toward reduced agonist efficacy and functional antagonism, identifying peptide 15 as the compound displaying the highest functional antagonist potency within this subset. Moreover, our strategy has improved resistance to proteolytic degradation for the analogues 6, 10, 14, and 15, highlighting the beneficial effect of macrocyclization on peptide stability. These results provide new insights into the interplay between structural modification and functional receptors responses, underscoring the complexity and potential of this research. This work establishes that, within 20-membered macrocycles, thioether bridges confer greater *h*MC4R selectivity than lactam bridges (peptide 14*vs.* peptides 6 and 8), and that cyclization topology can override dNal(2)-driven antagonism in 19-membered lactams (peptide 13). These findings provide a structural framework for designing next-generation melanocortin ligands with tailored *h*MC3R/*h*MC4R functional profiles.

## Experimental section

### Materials and general procedures

The *N*^α^-Fmoc-protected amino acids, such as Fmoc-Arg(Pbf)-OH, Fmoc-Asp-*O*All, Fmoc-His(Trt)-OH, Fmoc-Trp(Boc)-OH, and Fmoc-Cys(Trt)-OH, were purchased from IRIS Biotech GmbH (Marktredwitz, Germany). Unconventional amino acids, including Fmoc-β-Ala-OH, Fmoc-Dab(Alloc)-OH, Fmoc-Dap(Alloc)-OH, Fmoc-GABA-OH, Fmoc-dPhe-OH, and Fmoc-dNal(2)-OH, were obtained from GL Biochem Ltd (Shanghai, China) or Fluorochem (Hadfield, United Kingdom). Additional reagents such as 3-bromopropionic acid and 4-bromobutyric acid were commercially obtained from Merck Life Science (Milan, Italy). Activating and additive reagents such as *N*,*N*,*N*′,*N*′-tetramethyl-*O*-(1*H*-benzotriazol-1-yl)uranium hexafluorophosphate (HBTU), 1-hydroxybenzotriazole (HOBt), *N*,*N*′-diisopropylcarbodiimide (DIC), (7-azabenzotriazol-1-yloxy)trispyrrolidinophosphonium hexafluorophosphate (PyAOP), were obtained by GL Biochem Ltd (Shanghai, China) or Fluorochem (Hadfield, United Kingdom). The Fmoc-Rink amide resin (0.63 mmol g^−1^ of loading substitution), *N*,*N*-diisopropylethylamine (DIPEA), cesium carbonate (Cs_2_CO_3_), silver trifluoroacetate [Ag(CF_3_CO_2_)], piperidine, trifluoroacetic acid (TFA), and triisopropylsilane (TIS) were purchased from Merck Life Science (Milan, Italy). Additional reagents, including 1,3-dimethylbarbituric acid (NDMBA), tetrakis(triphenylphosphine)palladium(0) [Pd(PPh_3_)_4_], sodium *N*,*N*-diethyldithiocarbamate, and 4-dimethylaminopyridine (DMAP) were obtained from Merck Life Science (Milan, Italy). Solvents used for peptide synthesis, including *N*,*N*-dimethylformamide (DMF), dichloromethane (DCM), diethyl ether (Et_2_O), ethyl acetate (EtOAc), methanol (MeOH), as well as anhydrous solvents as DCM, DMF, and tetrahydrofuran (THF) were acquired from Merck Life Science (Milan, Italy). Solvents for HPLC analyses and purifications, such as water, and acetonitrile (MeCN), were of reagent grade acquired from commercial source (VWR, Milan, Italy) and used without further purification.

Purification of peptides was performed by RP-HPLC (Shimadzu Preparative Liquid Chromatograph LC-8A) equipped with a preparative column (Phenomenex Kinetex C18 column, 5 μm, 100 Å, 150 × 21.2 mm) using linear gradients of MeCN (0.1% TFA) in water (0.1% TFA), from 10 to 90% over 30 min, with a flow rate of 10 mL min^−1^ and UV detection at 220 nm. Final products were obtained by lyophilization of the appropriate fractions after removal of the MeCN by rotary evaporation. Analytical UHPLC (Shimadzu Nexera Liquid Chromatograph LC-30AD) analyses to evaluate the purity of peptides were performed on a Phenomenex Kinetex reversed-phase column (C18, 5 μm, 100 Å, 150 × 4.6 mm) with a flow rate of 1 mL min^−1^ using a gradient of MeCN (0.1% TFA) in water (0.1% TFA), from 10 to 90% over 15 min, and UV detection at 220 nm. All compounds examined for biological activity were purified to >97% (Fig. S1–S12), and the correct molecular ions were confirmed using ESI-MS (Fig. S13).

### Peptide synthesis

The synthesis was initiated using Fmoc-Rink amide resin (0.2 mmol for each sequence, 0.63 mmol g^−1^ as loading, 100–200 mesh as particle size). The resin was placed in a 10 mL polypropylene syringe tube (ISOLUTE® SPE filtration column by Biotage, Uppsala, Sweden), equipped with Teflon® filter, stopper, and stopcock, and pre-swollen in DMF for 30 min under agitation using an automated shaker (Multi Reax vortexer from Heidolph Instruments GmbH, Schwabach, Germany). Initial deprotection of the Fmoc-protecting group was accomplished using a 20% piperidine solution in DMF (5 min × 1, 25 min × 1). The first *N*^α^-Fmoc-protected amino acid (3 equiv.) was coupled using HBTU/HOBt (3 equiv. each), DIPEA (6 equiv.) in DMF. The coupling reaction proceeded for 2 h at rt on an automated shaker. Subsequent peptide elongation employed the US-SPPS method.^42^ Fmoc-deprotection steps involved treatment with 20% piperidine in DMF, coupled with ultrasonic irradiation (0.5 + 1 min). Amino acid coupling reactions used a solution of the *N*^α^-Fmoc-protected amino acid (3 equiv.), HBTU/HOBt (3 equiv. each), and DIPEA (6 equiv.) in DMF. Each coupling cycle involved 15 min under ultrasonic irradiation. Between steps, the resin underwent thorough washing with DMF (3 × 2 mL) and DCM (3 × 2 mL).^42^ Completion of Fmoc-deprotection and coupling reactions was monitored *via* Kaiser test used for the detection of solid-phase bound primary amines.

#### Lactam-bridged peptides (peptides 6–13)

*Head-to-side-chain* lactam cyclization of peptides 6, 7, 10, and 11 was performed on solid phase. According to previously reported procedures, the peptide sequences were subjected to selective removal of allyl-based protecting groups from Dab or Dap and aspartic acid residues.^43–45^ Briefly, the resin was washed with DCM (2 mL × 3), dried, and treated with a solution of Pd(PPh_3_)_4_ (0.15 equiv.) and NDMBA (3 equiv.) in dry DCM/DMF (3 : 2 v/v) and shaken for 1 h under shaking. The resin was filtered, washed with DMF (2 mL × 3) and DCM (2 mL × 3), dried, and the removal procedure was repeated once. The resin was subsequently washed with 0.5% sodium *N*,*N*-diethyldithiocarbamate solution in DMF for 30 min using an automated shaker. Complete removal of the allyl groups was confirmed by HPLC and MS analysis of the crude peptide obtained after cleavage of a resin aliquot [5 mg treated with 1 mL of TFA/TIS/H_2_O (95 : 2.5 : 2.5, v/v/v)]. Intramolecular lactam formation was then carried out by treating the resin with PyAOP (3 equiv.) and DIPEA (6 equiv.), dissolved in DMF, and the mixture was shaken for 12 h.^43,45^ Completion of the cyclization reaction was verified by HPLC and MS analysis of the crude peptide obtained after cleavage of a resin aliquot (5 mg). Finally, the N-terminal Fmoc group was removed as described above, and the peptidyl resin was washed with DCM (2 mL × 3), and dried under reduced pressure.

For peptides 8, 9, 12, and 13 the lactam bridge involves a GABA residue (peptides 8 and 9) or a β-Ala residue (peptides 12 and 13) and aspartic acid residue. Specifically, the resin was first treated for the removal of the allyloxycarbonyl protecting group from the aspartic acid residue, as described above.^43,45^ Subsequently, it was treated for Fmoc-removal from GABA or β-Ala was performed as described previously. The released amine and carboxylic acid were coupled using PyAOP (3 equiv.) and DIPEA (6 equiv.) in DMF, and the mixture was shaken on an automated shaker for 12 h. The coupling reaction was monitored by HPLC and MS analysis of the crude peptide obtained after cleavage of a resin aliquot (5 mg). Finally, the peptidyl resin was washed with DCM (2 mL × 3) and dried *in vacuo*.

#### Thioether-bridged peptides

For peptides 14–17, the thioether cyclization was achieved off-line.^48^ In detail, after Fmoc removal from His residue, 4-bromobutyric acid (peptides 14 and 15) or 3-bromopropionic acid (peptides 16 and 17) (2 equiv.) was preactivated with DIC (1 equiv.) in dry THF. The reaction mixture was shaken on an automated shaker for 15 min to allow preactivation.^46,47^ The activated mixture was then added to the resin and allowed to react for 12 h. Successful coupling was confirmed by HPLC and MS analysis of the crude peptide obtained after cleavage of a resin aliquot (5 mg). Subsequently, the resin was washed with DCM (2 mL × 3), dried *in vacuo*, and cleaved using a cocktail of TFA/TIS/H_2_O (95 : 2.5 : 2.5, v/v/v) for 3 h at rt. The crude peptide (0.035 mmol, purity >85%) was dissolved in H_2_O/MeCN (1 : 1, v/v), at a final concentration 1.2 mM. DMAP (0.3 equiv.) was then added, and the solution was stirred for 10 min. Subsequently, DIPEA (3 equiv.) was added, and the reaction mixture was stirred for 12 h at rt.^48^ Afterwards, MeCN was removal by rotary evaporation, and the crude peptide was lyophilized. Successful cyclization was confirmed by HPLC and MS analysis. Interestingly, peptides 16 and 17 were also obtained through an alternative on-resin cyclization strategy. In detail, the peptidyl resin was swollen in DCM for 20 min and subject to *S*-detritylation on solid phase using Ag(CF_3_CO_2_) (0.4 equiv.) in DCM (3 mL). The mixture was agitated on an automated shaker for 15 min, as reported elsewhere.^49^ Subsequently, the resin was washed with DMF/MeOH (1 : 1, v/v, 3 × 2 mL), EtOAc (3 × 2 mL), and DCM (3 × 2 mL), and the *S*-detritylation reaction was repeated for a second cycle. Finally, the peptidyl resin was treated with Cs_2_CO_3_ (4 equiv.) in dry DMF and shaken for 12 h at rt.^49^ The resin was then washed with MeOH/DMF (1 : 1, v/v, 2 mL × 3), DMF/H_2_O (8 : 2, v/v, 2 mL × 3), EtOAc (2 mL × 3), and DCM (2 mL × 3), and dried under *vacuum*. Successful cyclization was monitored by HPLC and MS analysis of the crude peptide obtained after cleavage of a resin aliquot (5 mg).

#### Peptide cleavage

All resin-bound peptides deblocked from the N-terminal Fmoc group were thoroughly washed with DCM (2 mL × 3) and dried. Subsequently, they were released from the resin, and the protecting groups were cleaved simultaneously by treatment with a cocktail of TFA/TIS/H_2_O (95 : 2.5 : 2.5 v/v/v) for 3 h at rt. The resin was then removed by filtration, and the crude peptides were recovered by precipitation with chilled Et_2_O twice and separated by centrifugation (15 min × 2, 600 rpm). The supernatants were removed, the crude products were dried under *vacuum* and then dissolved in 10% MeCN in H_2_O for purification by RP-HPLC.

### Circular dichroism spectroscopy

The secondary structure of all peptides was studied in water by circular dichroism (CD) spectroscopy. Each peptide was dissolved in water at 200 μM and CD spectrum was recorded from 195 to 260 nm using a Jasco J-1500 CD Spectrometer with a quartz cell (0.1 cm) at room temperature. Each spectrum was obtained by averaging three scans and converting the signal to molar ellipticity.

### Pharmacological assays

#### cAMP response assays

Peptide activity was characterized using clonal HEK293 cells stably transfected with *h*MC3R or *h*MC4R and the GloSensor™-22F sensor (Promega, Madison, WI) to quantify intracellular cAMP levels in response to ligand exposure. Assays were conducted at room temperature in triplicate to sextuplicate, and EC_50_ and IC_50_ values were determined by nonlinear regression using a four-parameter logistic (sigmoid) model in GraphPad Prism 11 (San Diego, CA). Each assay plate included a control α-MSH concentration–response curve to normalize the test peptide maximum responses (*E*_max_). Luminescence from the GloSensor was measured in real time over fifteen-minute windows in the absence (agonist mode) and presence (antagonist mode) of an EC_90_-equivalent concentration of α-MSH using a Hamamatsu FDSS 7000EX plate reader. Activity at each concentration was determined by area-under-the-curve analysis. A detailed description of the method has been published elsewhere.^54^

To define the functional characteristics of the peptides described herein, we used the following criteria: compounds with at least 90% efficacy relative to the reference standard agonist were classified as full agonists, while compounds eliciting a response of ≤10% above baseline were classified as full antagonists. Peptides with maximal responses between 10% and 90% were designated as partial agonists; those with efficacy below 50% were classified as weak partial agonists.

### Human serum stability assays

Peptide stability was evaluated by modifying previously described procedures.^45^ The assays were performed in human serum. For human serum stability, 2 mM peptide stock solutions were prepared in water and mixed with human serum (from male AB plasma, USA origin, sterile-filtered, Merck/Sigma-Aldrich, USA) to obtain a final concentration of 200 μM [human plasma/stock solution (9 : 1, v/v)]. The mixtures were incubated at 37 ± 1 °C and 50 μL aliquots were taken at predetermined time intervals (0,2, 4, 8 and 24 h). Protease activity was quenched by adding 100 μL of acetonitrile (MeCN). The resulting cloudy suspensions were cooled t 4 °C for 10 min and centrifugated at 12 000 rpm for 10 min to remove the serum proteins. Then, the supernatants were analyzed by analytical HPLC (Shimadzu Nexera Liquid Chromatograph LC-30AD) which was equipped with a Phenomenex Kinetex reversed-phase column (C18, 5 μm, 100 Å, 150 mm × 4.6 mm) with a flow rate of 1 mL min^−1^ using a gradient of MeCN (0.1%TFA) in water (0.1%TFA), from 10 to 90% over 15 min, and UV detection at 220 nm. All experiments were conducted in triplicate. The percentage of intact peptides at each time point was determined by integrating the corresponding HPLC peak areas.

## Conflicts of interest

R. D. C., L. E. G., S. Y. W., and the University of Michigan have equity in Courage Therapeutics, Inc.; L. E. G. is an employee of Courage Therapeutics, Inc., and R. D. C. serves on the company board. The other authors declare no competing interests.

## Supplementary Material

MD-OLF-D6MD00726K-s001

## Data Availability

The data supporting this article have been included as part of the supplementary information (SI). Supplementary information: the SI includes analytical data, HPLC chromatograms and MS spectra for cyclic peptides 6–17, concentration-response profiles for cAMP accumulation, and the predicted conformation of peptide 6. See DOI: https://doi.org/10.1039/d6md00726k.
