# Supplementary material for: Downsizing macrocycles in melanocortin peptide analogues: thioether and lactam cyclization strategies to modulate hMC3/4R agonism and antagonism
Source: RSC Med Chem. 2026 Oct 21. Online ahead of print. doi: 10.1039/d6md00726k (PMC13736795; doi:10.1039/d6md00726k)
Supplement: Supplementary file 1 [file MD-OLF-D6MD00726K-s001.pdf]

Supplementary Information (SI) for RSC Medicinal Chemistry.
This journal is © The Royal Society of Chemistry

Supporting information

Downsizing Macrocycles in Melanocortin Peptide Analogues: Thioether and Lactam Cyclization Strategies to Modulate *h*MC3/4R Agonism and Antagonism

Rosa Bellavita,[a] Luis E. Gimenez,[b] Savannah Y. Williams,[b] Ida Boccino,[a] Gennaro M. Bailo,[a] Roger D. Cone,[b,c,d] Paolo Grieco[a] and Francesco Merlino[a,]*

[a]Department of Pharmacy, University of Naples Federico II, Naples 80131, Italy

[b]Life Sciences Institute, University of Michigan, Ann Arbor, MI 48109, USA

[c]Department of Molecular and Integrative Physiology, University of Michigan, Ann Arbor, MI 48109, USA

[d]Department of Molecular, Cellular, and Developmental Biology, University of Michigan, Ann Arbor, MI 48109, USA

Table of contents

1. Analytical data of cyclic peptides **6-17** (Table S1) S2
2. HPLC chromatograms of cyclic peptides **6-17** (Figures S1-12) S3
3. MS spectra of cyclic peptides **6-17** (Figure S13) S9
4. Concentration-response profiles for cAMP accumulation (Table S2) S11
5. Predicted conformation of peptide **6** (Figure S14) S12

1. **Table S1**. *Analytical data for cyclic peptides (**6-17**)*

ID	t_R (min)	Molecular Formula	$[M+H]^+_{calcd}$	$[(M+nH)/n]^+_{obs}$
**RB-91 (6)**	9.3	$C_{40}H_{53}N_{14}O_7^+$	841.4	841.1, n=1<br>421.4, n=2
**RB-90 (7)**	10.2	$C_{44}H_{55}N_{14}O_7^+$	891.4	891.4, n=1<br>446.5, n=2
**RB-94 (8)**	9.9	$C_{40}H_{52}N_{13}O_7^+$	826.4	826.4, n=1<br>413.9, n=2
**RB-95 (9)**	10.7	$C_{44}H_{54}N_{13}O_7^+$	876.4	876.4, n=1<br>438.9, n=2
**RB-89 (10)**	9.2	$C_{39}H_{51}N_{14}O_7^+$	827.4	827.4, n=1<br>414.4, n=2
**RB-92 (11)**	10.1	$C_{43}H_{53}N_{14}O_7^+$	877.4	877.4 n=1<br>439.4, n=2
**RB-96 (12)**	9.6	$C_{39}H_{50}N_{13}O_7^+$	812.4	812.4, n=1<br>406.9, n=2
**RB-97 (13)**	10.5	$C_{43}H_{52}N_{13}O_7^+$	862.4	862.4, n=1<br>431.9, n=2
**RB-88 (14)**	9.9	$C_{39}H_{51}N_{12}O_6S^+$	815.4	815.5 n=1
**RB-93 (15)**	10.8	$C_{43}H_{53}N_{12}O_6S^+$	865.4	865.5, n=1
**RB-84 (16)**	9.8	$C_{38}H_{49}N_{12}O_6S^+$	801.4	801.4, n=1<br>401.2, n=2
**RB-85 (17)**	10.7	$C_{42}H_{51}N_{12}O_6S^+$	851.4	851.4 n=1<br>426.5, n=2

2. HPLC chromatograms of peptides ***6-17***

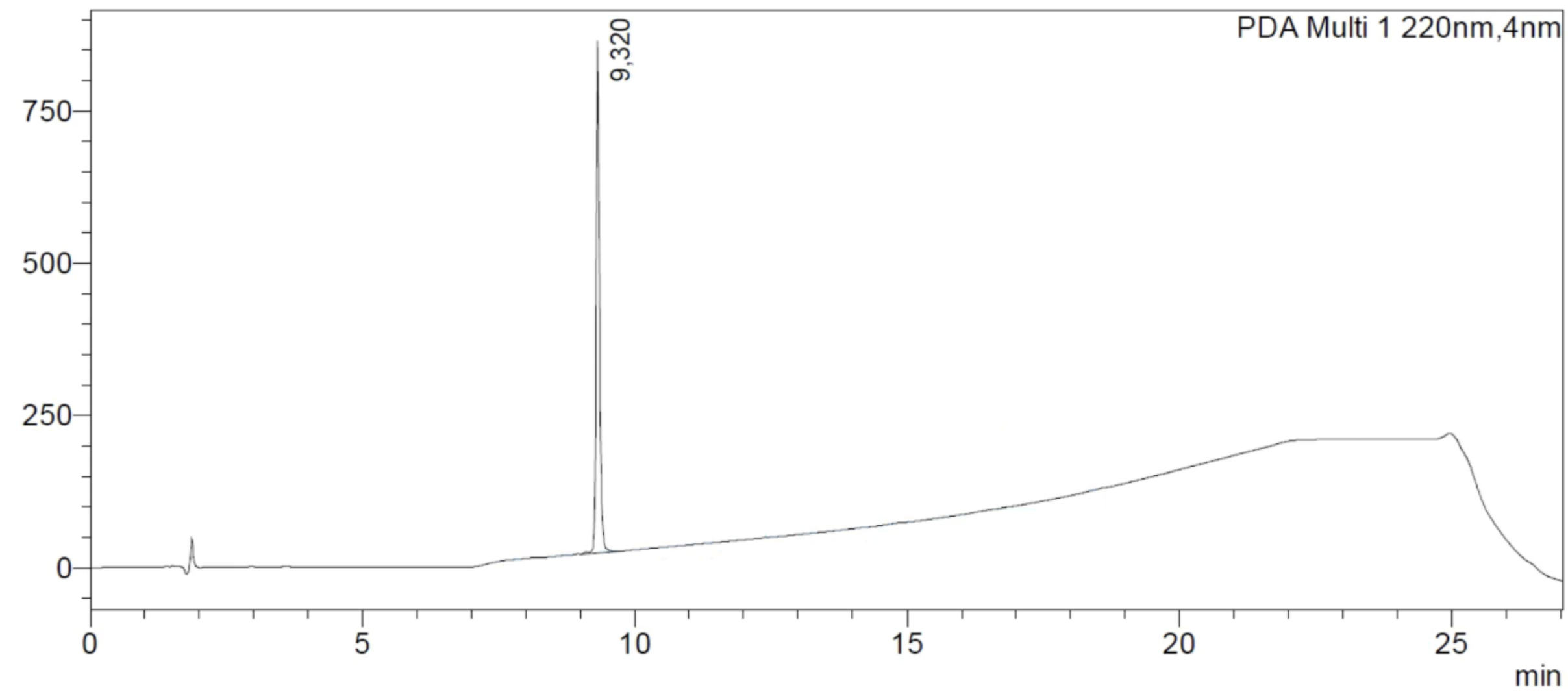

Figure S1. Chromatogram of **RB-91** (**6**) obtained by an analytical HPLC (Shimadzu UFLC SPD-M20A/DGU-20A3R/LC-20AD) equipped with a Phenomenex Kinetex C18 column (150 mm × 4.6 mm, 5 μm, 100 Å), and monitored by UV detection at 220 nm. t_R= 9.3 min [linear gradient 10-90% MeCN (0.1% TFA) in H_2O (0.1% TFA) over 15 min, flow rate of 1 mL/min].

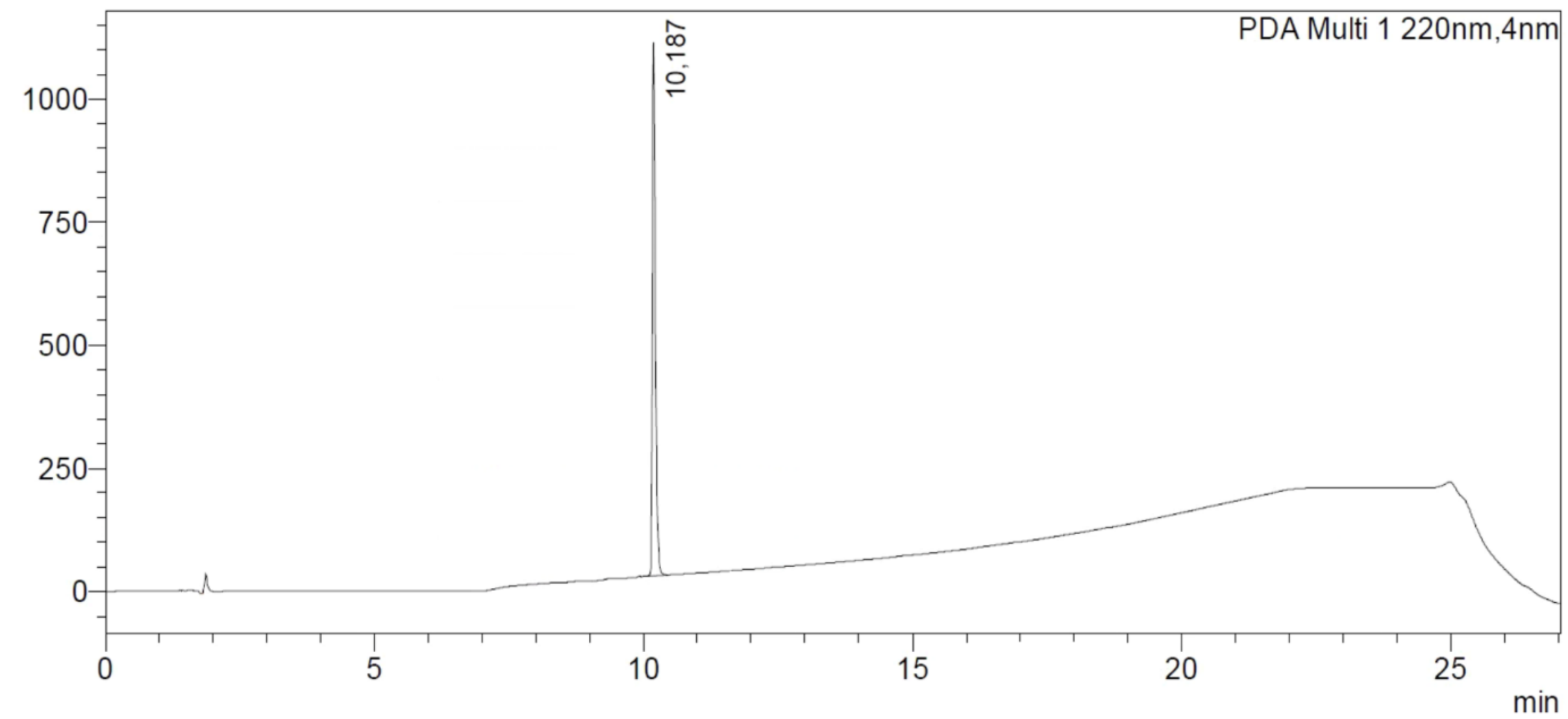

Figure S2. Chromatogram of **RB-90** (**7**) obtained by an analytical HPLC (Shimadzu UFLC SPD-M20A/DGU-20A3R/LC-20AD) equipped with a Phenomenex Kinetex C18 column (150 mm × 4.6 mm, 5 μm, 100 Å), and monitored by UV detection at 220 nm. t_R= 10.2 min [linear gradient 10-90% MeCN (0.1% TFA) in H_2O (0.1% TFA) over 15 min, flow rate of 1 mL/min].

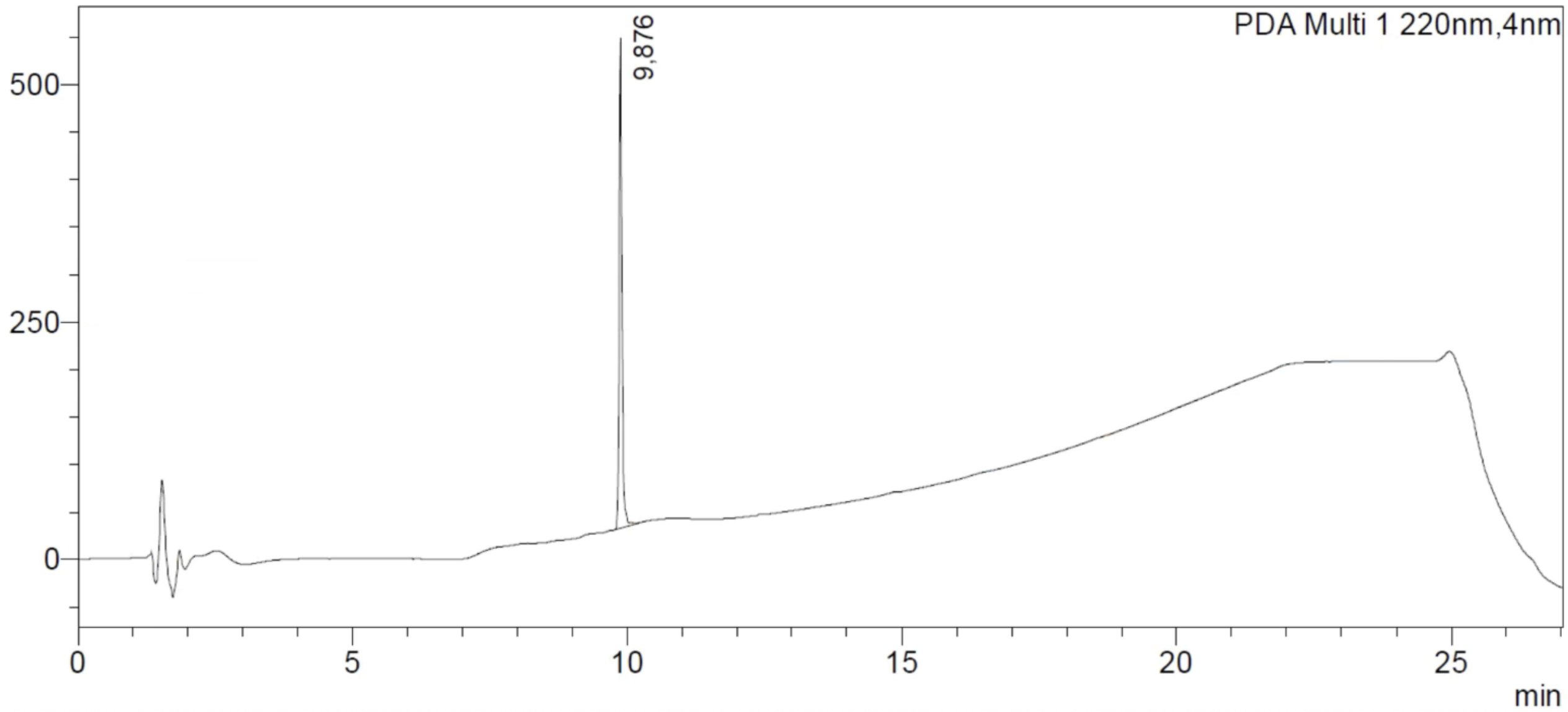

Figure S3. Chromatogram of **RB-94** (**8**) obtained by an analytical HPLC (Shimadzu UFLC SPD-M20A/DGU-20A3R/LC-20AD) equipped with a Phenomenex Kinetex C18 column (150 mm × 4.6 mm, 5 μm, 100 Å), and monitored by UV detection at 220 nm. t_R= 9.9 min [linear gradient 10-90% MeCN (0.1% TFA) in H_2O (0.1% TFA) over 15 min, flow rate of 1 mL/min].

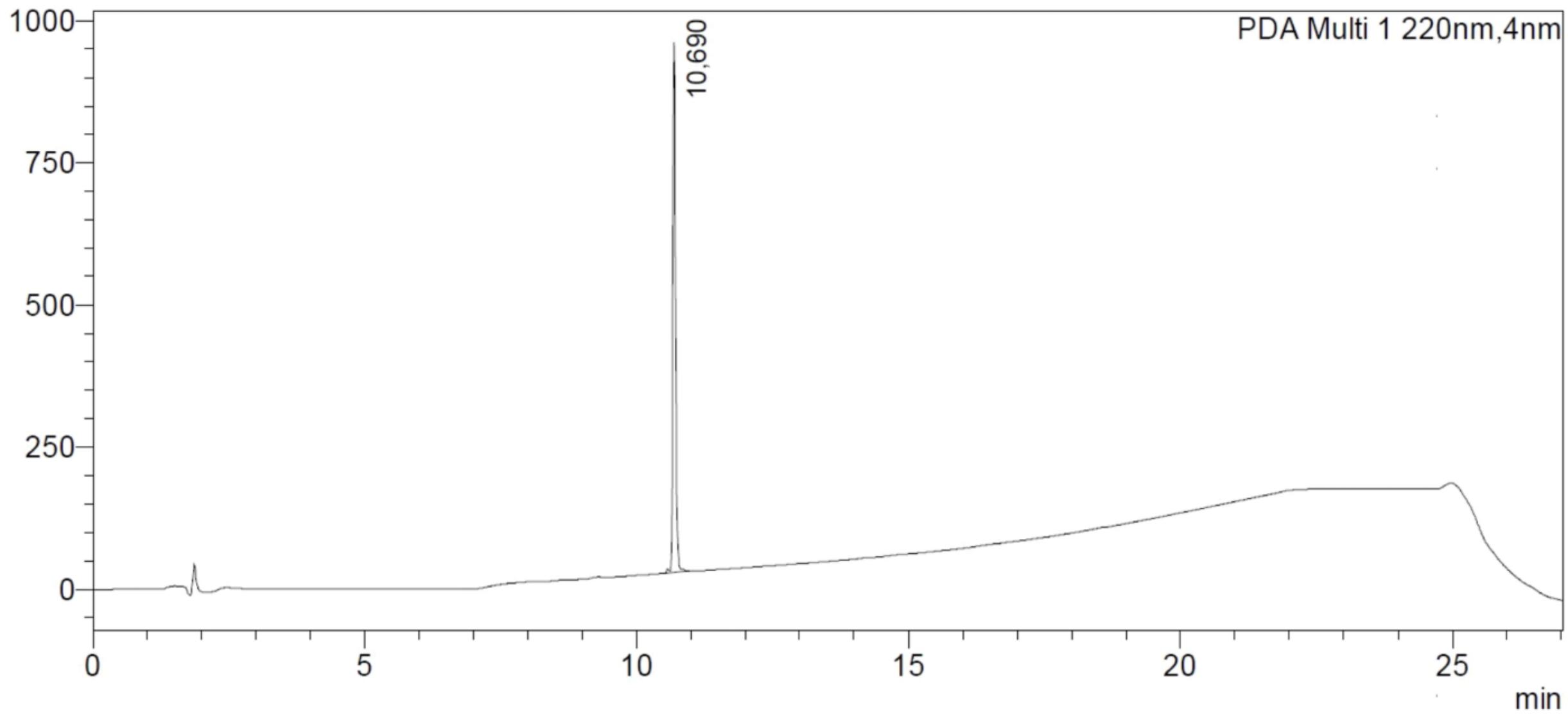

Figure S4. Chromatogram of **RB-95** (**9**) obtained by an analytical HPLC (Shimadzu UFLC SPD-M20A/DGU-20A3R/LC-20AD) equipped with a Phenomenex Kinetex C18 column (150 mm × 4.6 mm, 5 μm, 100 Å), and monitored by UV detection at 220 nm. t_R= 10.7 min [linear gradient 10-90% MeCN (0.1% TFA) in H_2O (0.1% TFA) over 15 min, flow rate of 1 mL/min].

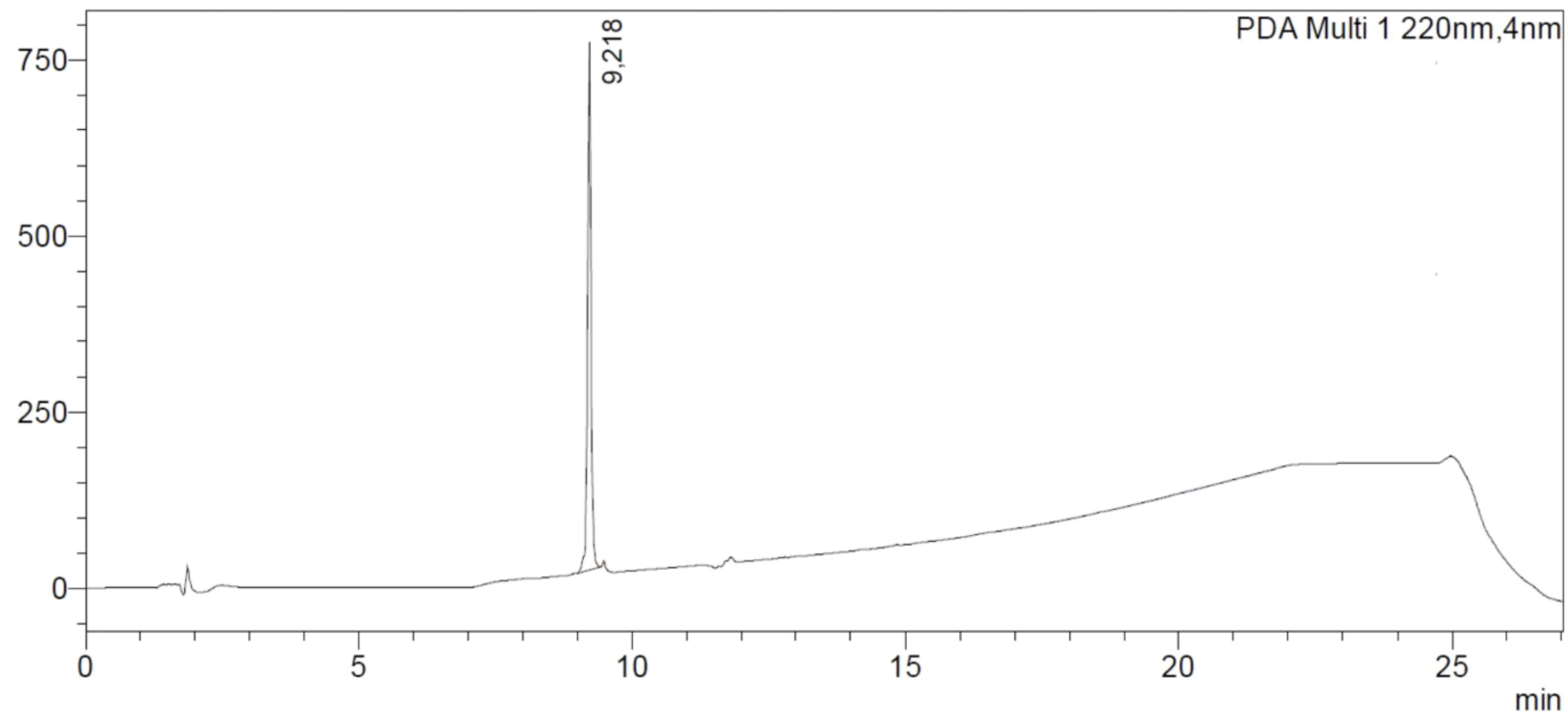

Figure S5. Chromatogram of **RB-89 (10)** obtained by an analytical HPLC (Shimadzu UFLC SPD-M20A/DGU-20A3R/LC-20AD) equipped with a Phenomenex Kinetex C18 column (150 mm × 4.6 mm, 5 μm, 100 Å), and monitored by UV detection at 220 nm. t_R= 9.2 min [linear gradient 10-90% MeCN (0.1% TFA) in H_2O (0.1% TFA) over 15 min, flow rate of 1 mL/min].

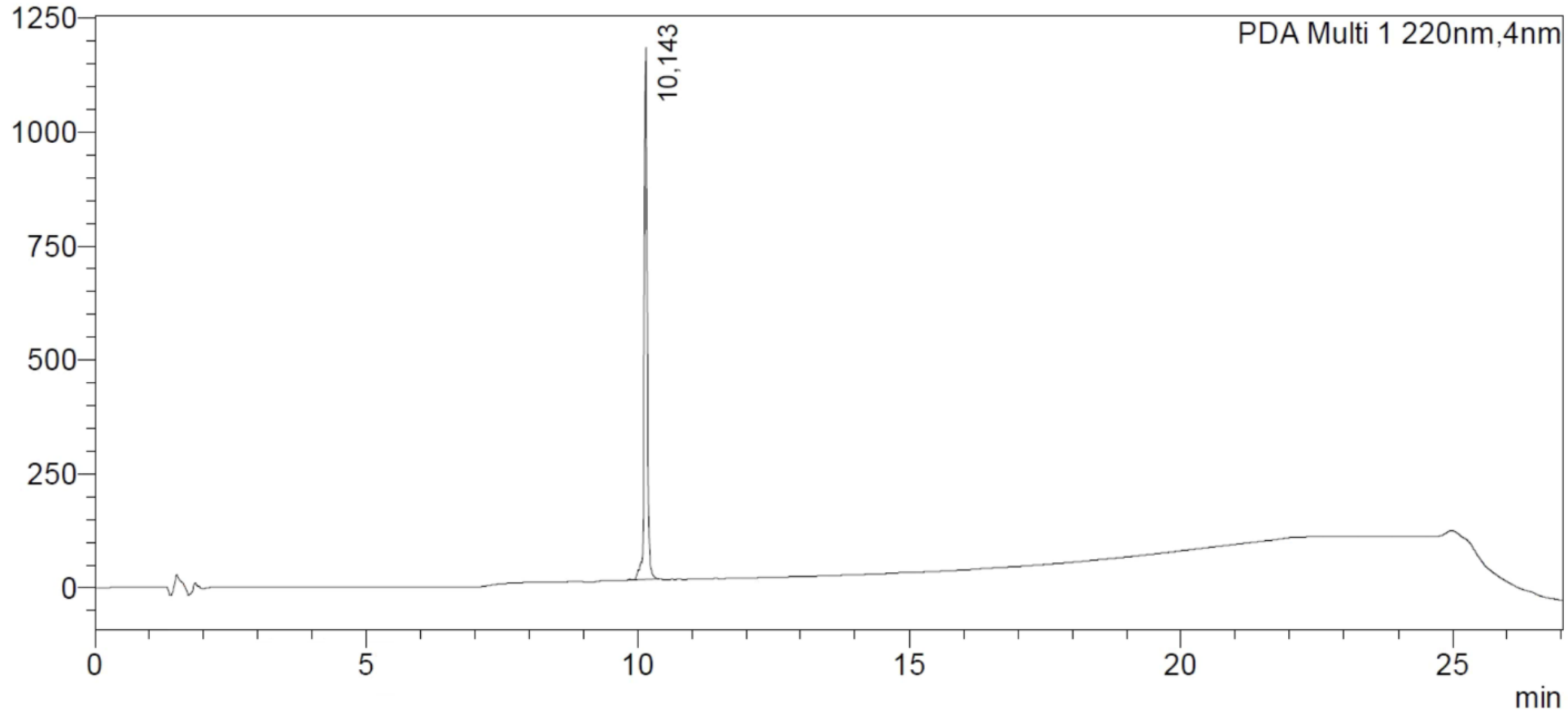

Figure S6. Chromatogram of **RB-92 (11)** obtained by an analytical HPLC (Shimadzu UFLC SPD-M20A/DGU-20A3R/LC-20AD) equipped with a Phenomenex Kinetex C18 column (150 mm × 4.6 mm, 5 μm, 100 Å), and monitored by UV detection at 220 nm. t_R= 10.1 min [linear gradient 10-90% MeCN (0.1% TFA) in H_2O (0.1% TFA) over 15 min, flow rate of 1 mL/min].

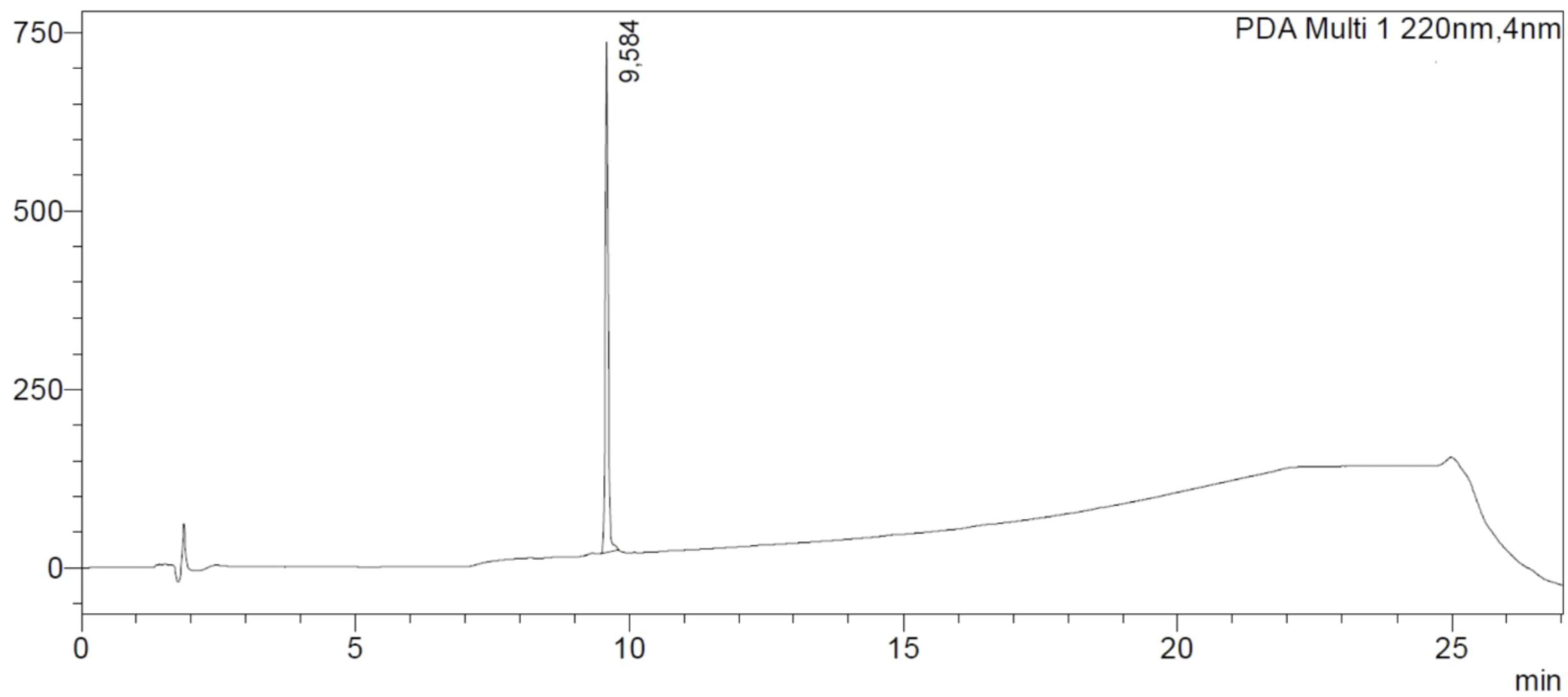

Figure S7. Chromatogram of **RB-96 (12)** obtained by an analytical HPLC (Shimadzu UFLC SPD-M20A/DGU-20A3R/LC-20AD) equipped with a Phenomenex Kinetex C18 column (150 mm × 4.6 mm, 5 μm, 100 Å), and monitored by UV detection at 220 nm. t_R= 9.6 min [linear gradient 10-90% MeCN (0.1% TFA) in H_2O (0.1% TFA) over 15 min, flow rate of 1 mL/min].

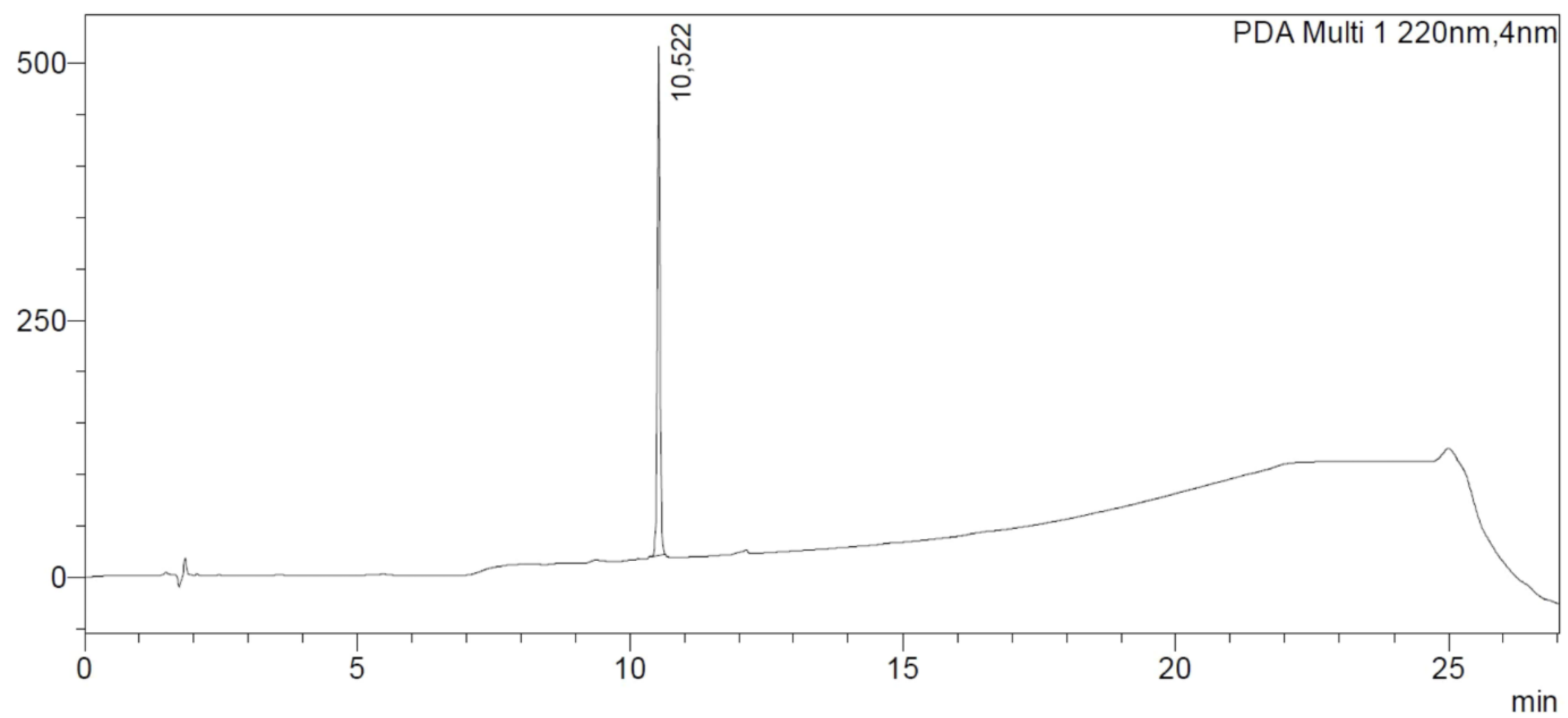

Figure S8. Chromatogram of **RB-97 (13)** obtained by an analytical HPLC (Shimadzu UFLC SPD-M20A/DGU-20A3R/LC-20AD) equipped with a Phenomenex Kinetex C18 column (150 mm × 4.6 mm, 5 μm, 100 Å), and monitored by UV detection at 220 nm. t_R= 10.5 min [linear gradient 10-90% MeCN (0.1% TFA) in H_2O (0.1% TFA) over 15 min, flow rate of 1 mL/min].

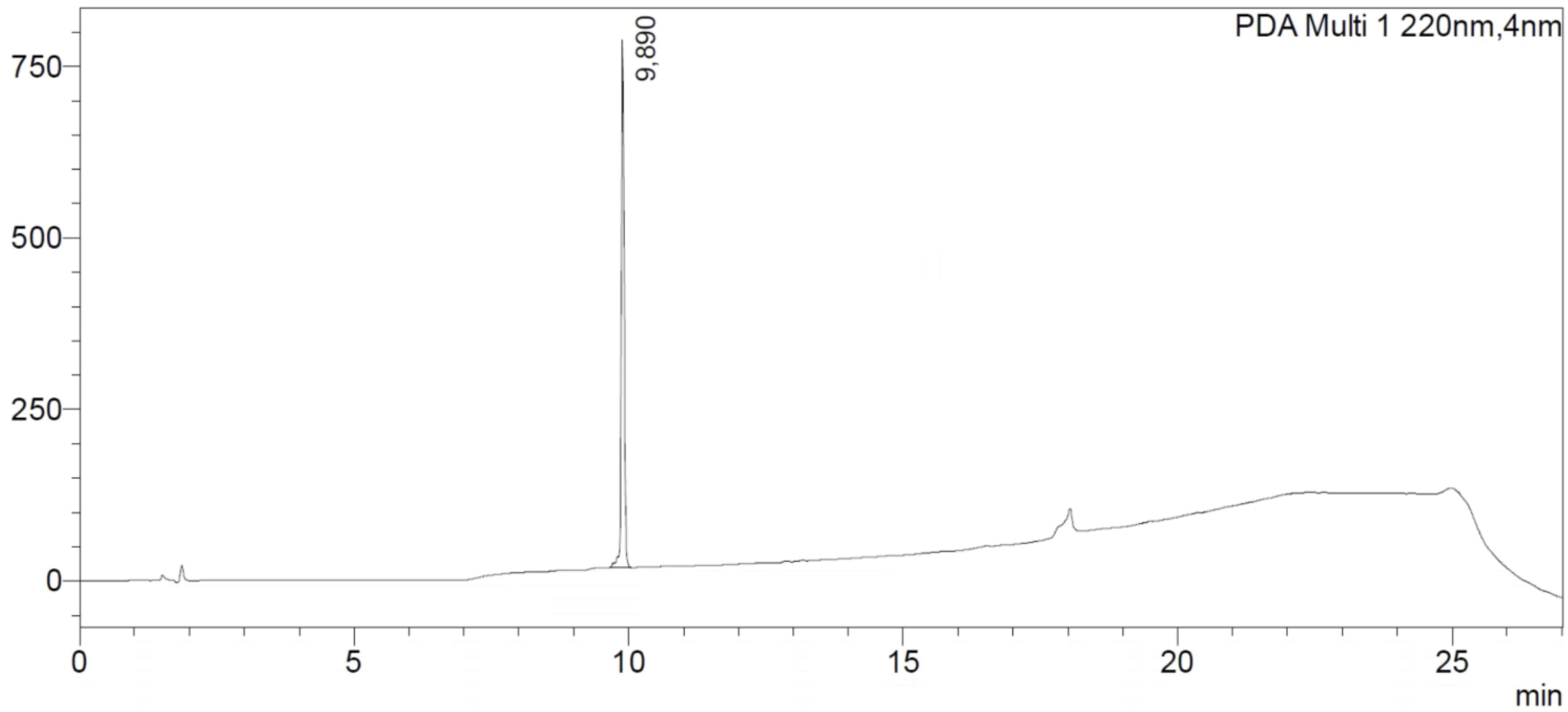

Figure S9. Chromatogram of **RB-88 (14)** obtained by an analytical HPLC (Shimadzu UFLC SPD-M20A/DGU-20A3R/LC-20AD) equipped with a Phenomenex Kinetex C18 column (150 mm × 4.6 mm, 5 μm, 100 Å), and monitored by UV detection at 220 nm. t_R= 9.9 min [linear gradient 10-90% MeCN (0.1% TFA) in H_2O (0.1% TFA) over 15 min, flow rate of 1 mL/min].

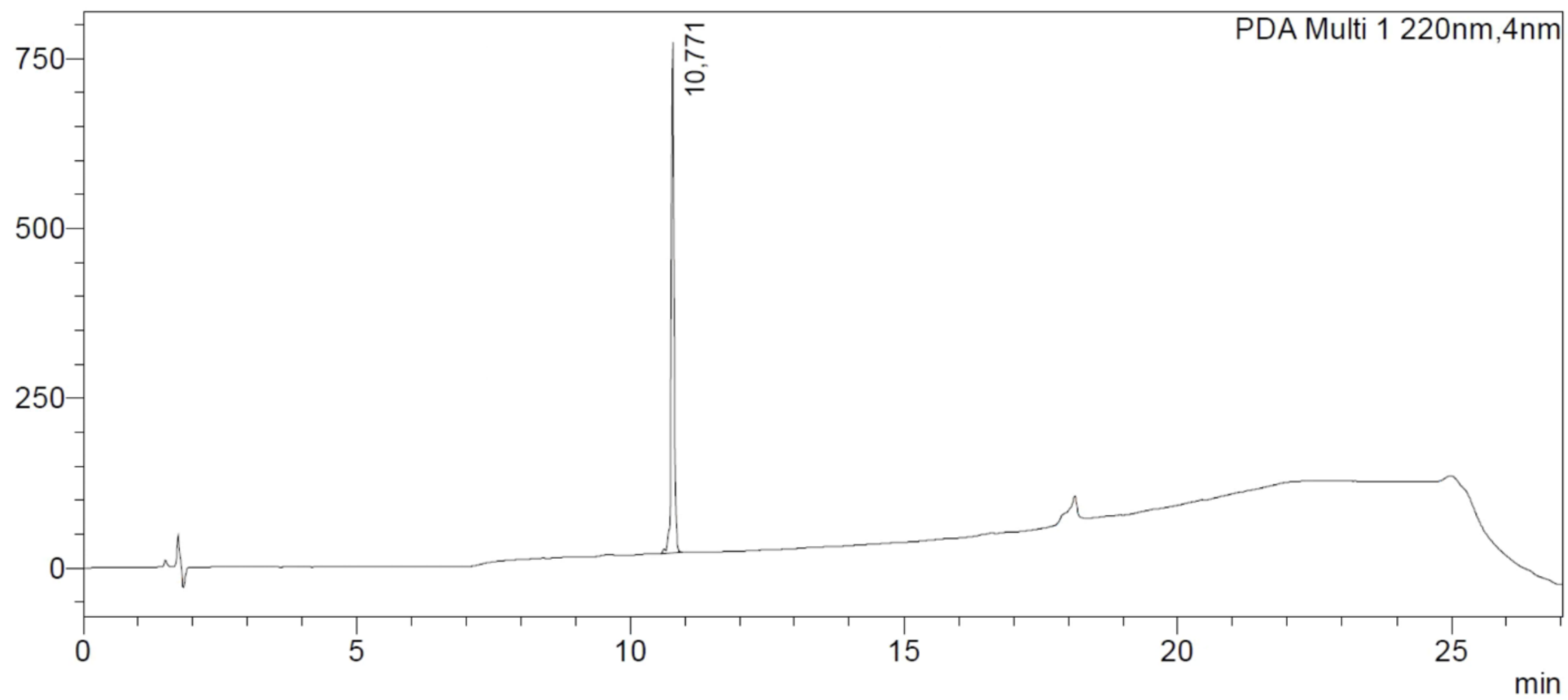

Figure S10. Chromatogram of **RB-93 (15)** obtained by an analytical HPLC (Shimadzu UFLC SPD-M20A/DGU-20A3R/LC-20AD) equipped with a Phenomenex Kinetex C18 column (150 mm × 4.6 mm, 5 μm, 100 Å), and monitored by UV detection at 220 nm. t_R= 10.8 min [linear gradient 10-90% MeCN (0.1% TFA) in H_2O (0.1% TFA) over 15 min, flow rate of 1 mL/min].

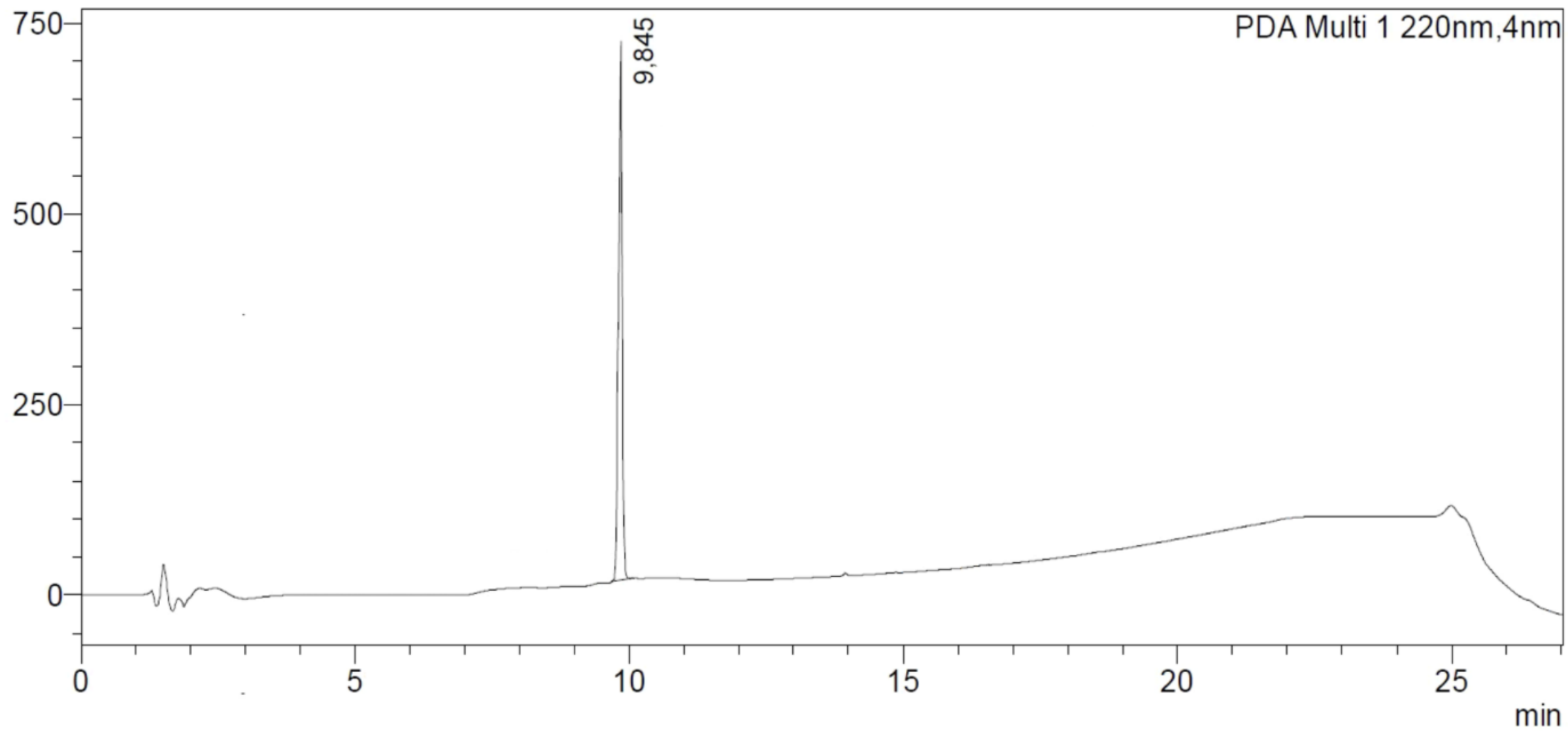

Figure S11. Chromatogram of **RB-84 (16)** obtained by an analytical HPLC (Shimadzu UFLC SPD-M20A/DGU-20A3R/LC-20AD) equipped with a Phenomenex Kinetex C18 column (150 mm × 4.6 mm, 5 μm, 100 Å), and monitored by UV detection at 220 nm. t_R= 9.8 min [linear gradient 10-90% MeCN (0.1% TFA) in H_2O (0.1% TFA) over 15 min, flow rate of 1 mL/min].

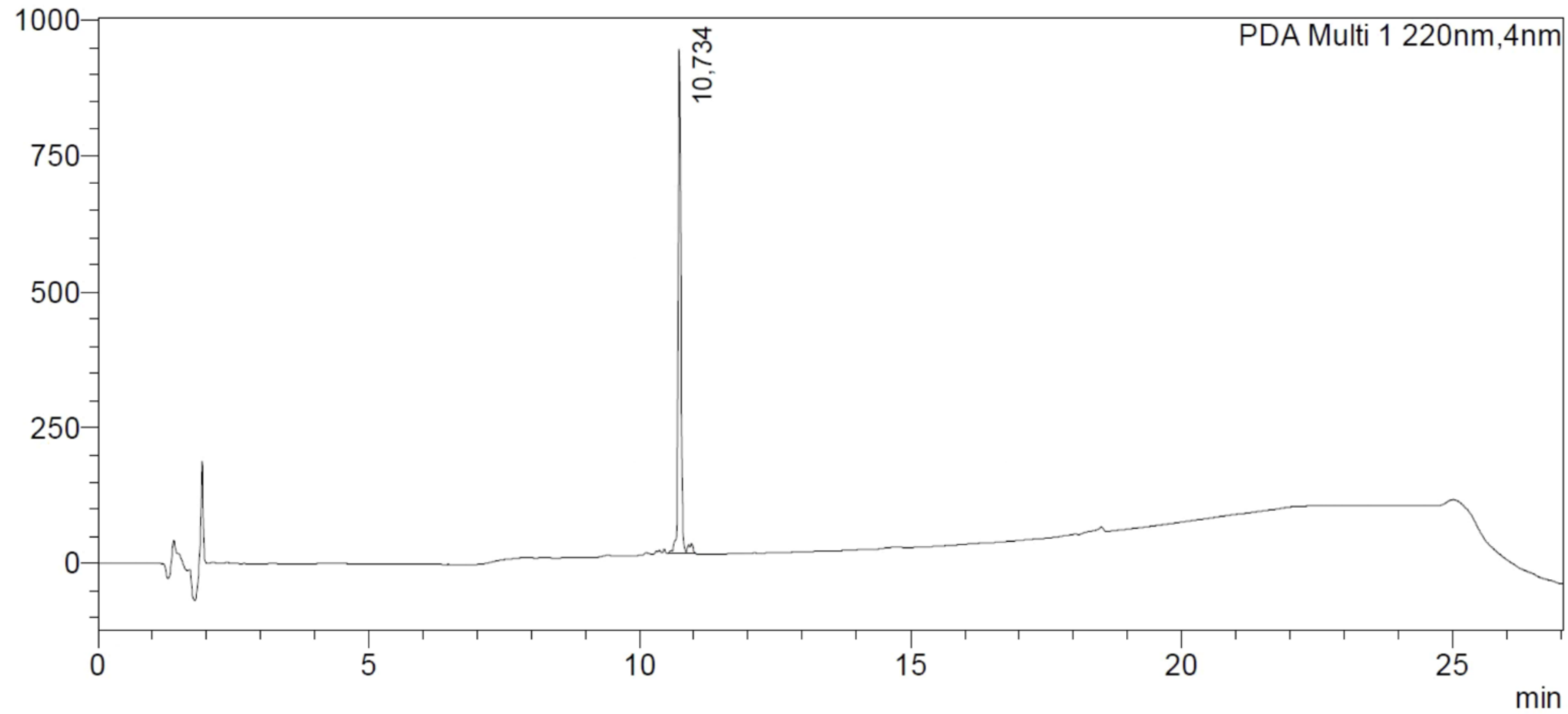

Figure S12. Chromatogram of **RB-85 (17)** obtained by an analytical HPLC (Shimadzu UFLC SPD-M20A/DGU-20A3R/LC-20AD) equipped with a Phenomenex Kinetex C18 column (150 mm × 4.6 mm, 5 μm, 100 Å), and monitored by UV detection at 220 nm. t_R= 10.7 min [linear gradient 10-90% MeCN (0.1% TFA) in H_2O (0.1% TFA) over 15 min, flow rate of 1 mL/min].

*3. MS spectra of cyclic peptides (**6-17**)*

(A)

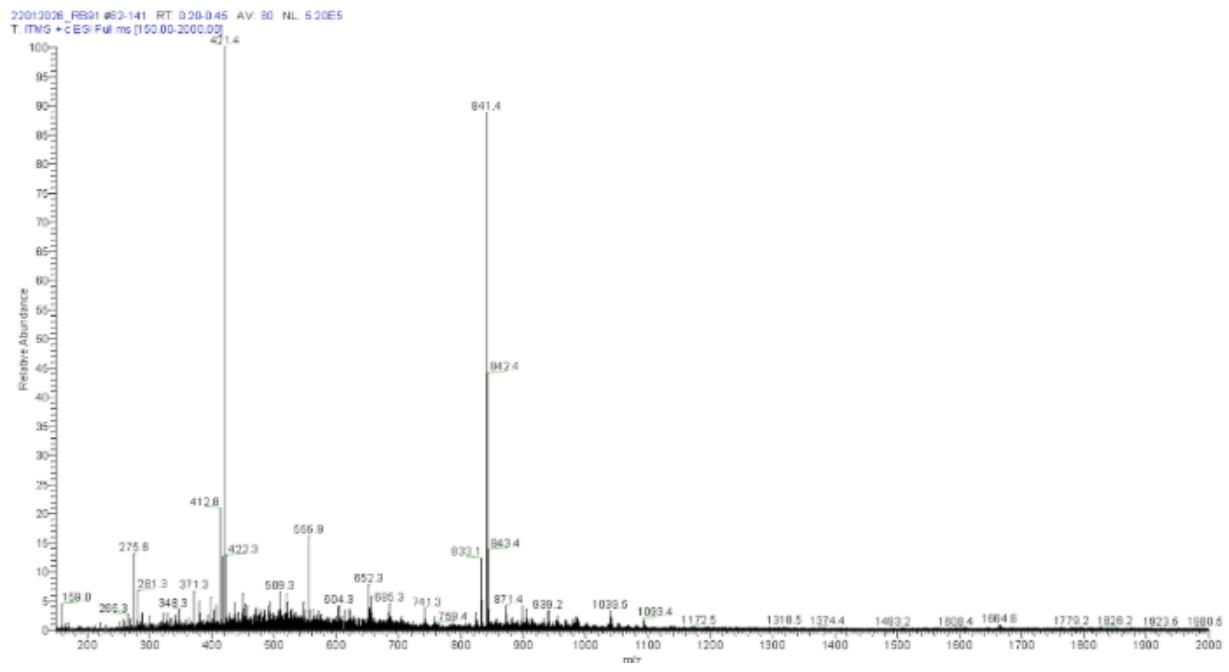

(B)

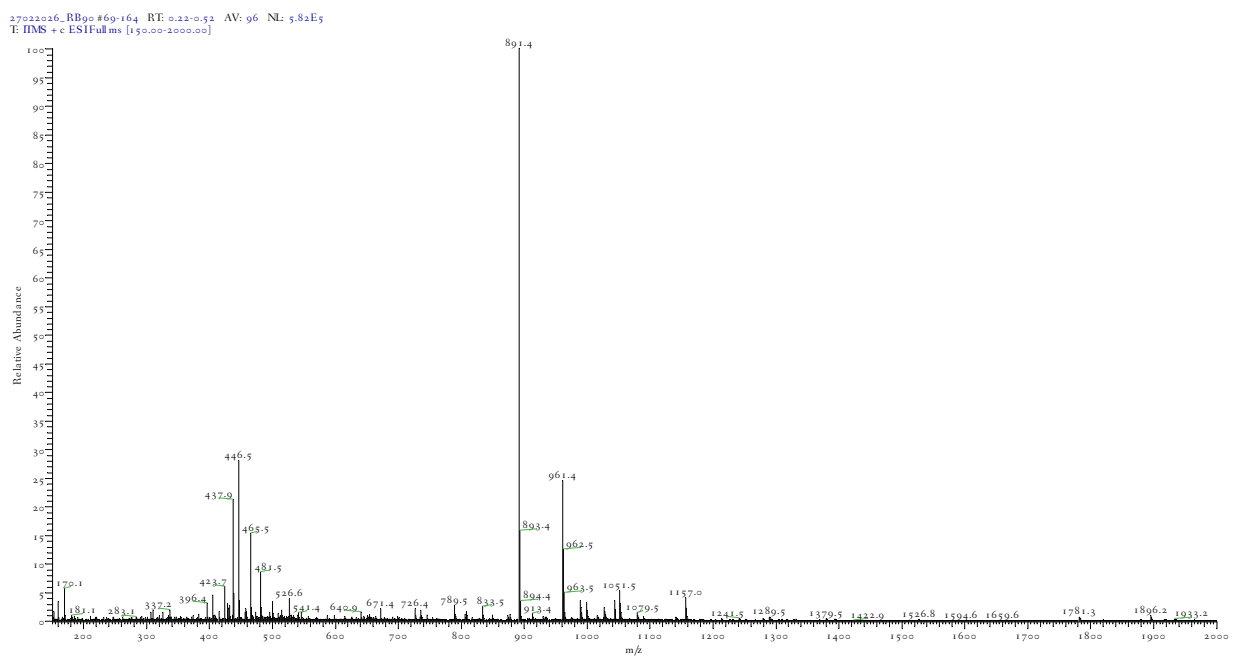

(C)

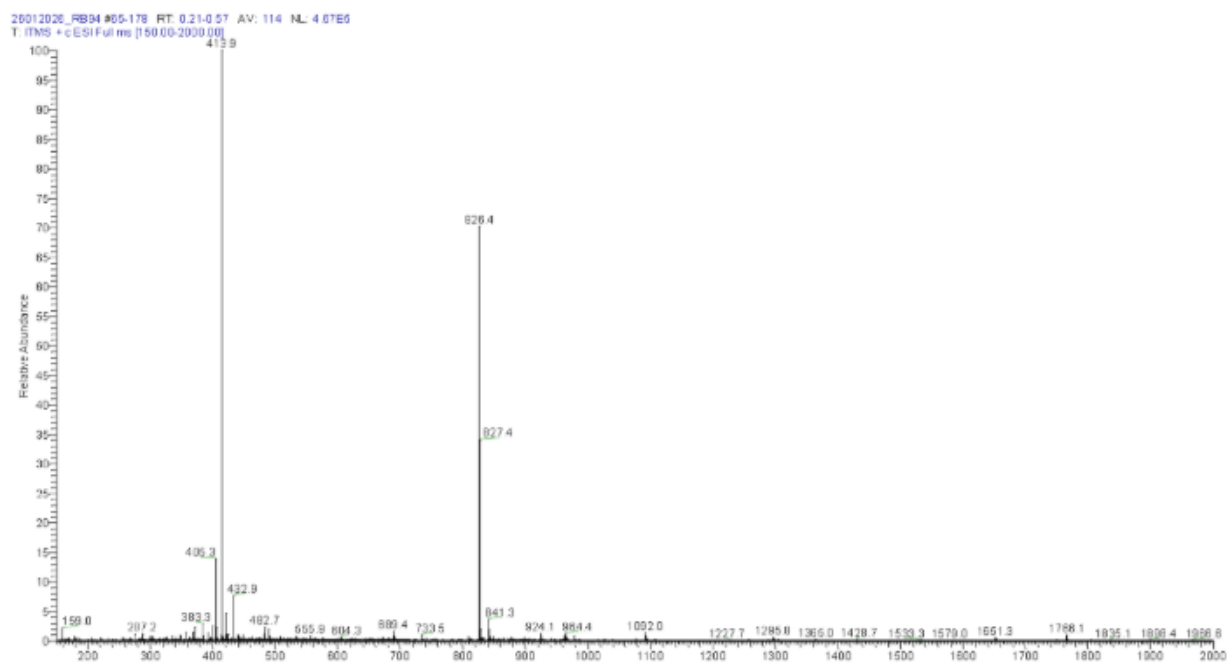

(D)

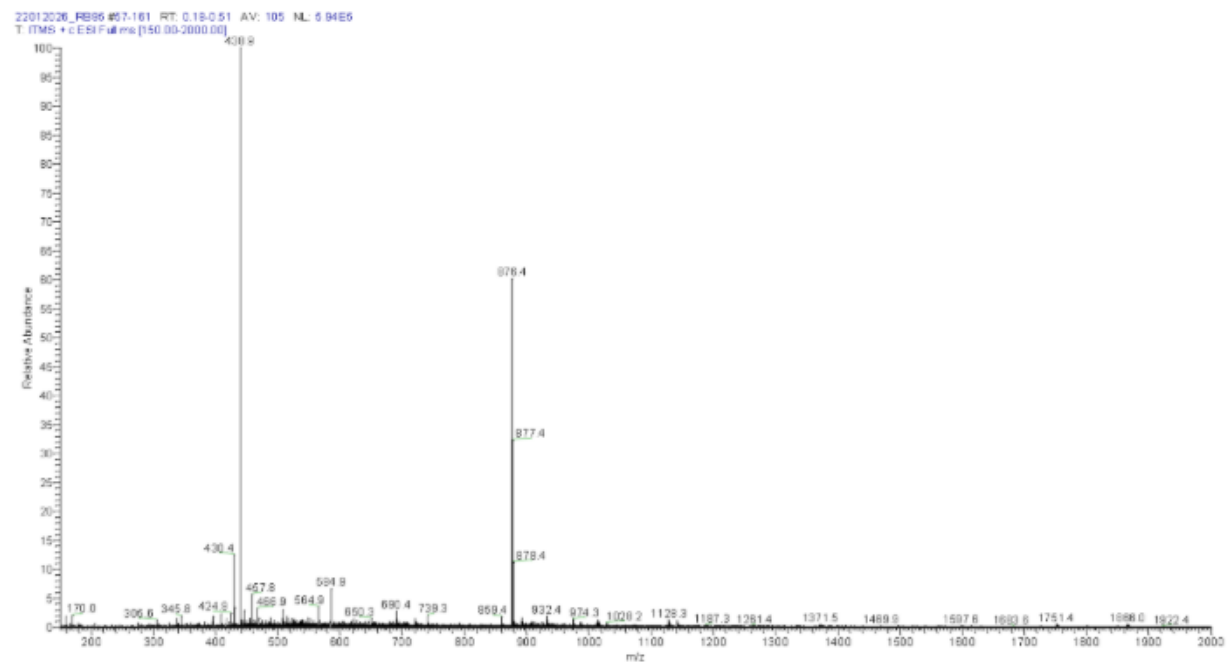

(E)

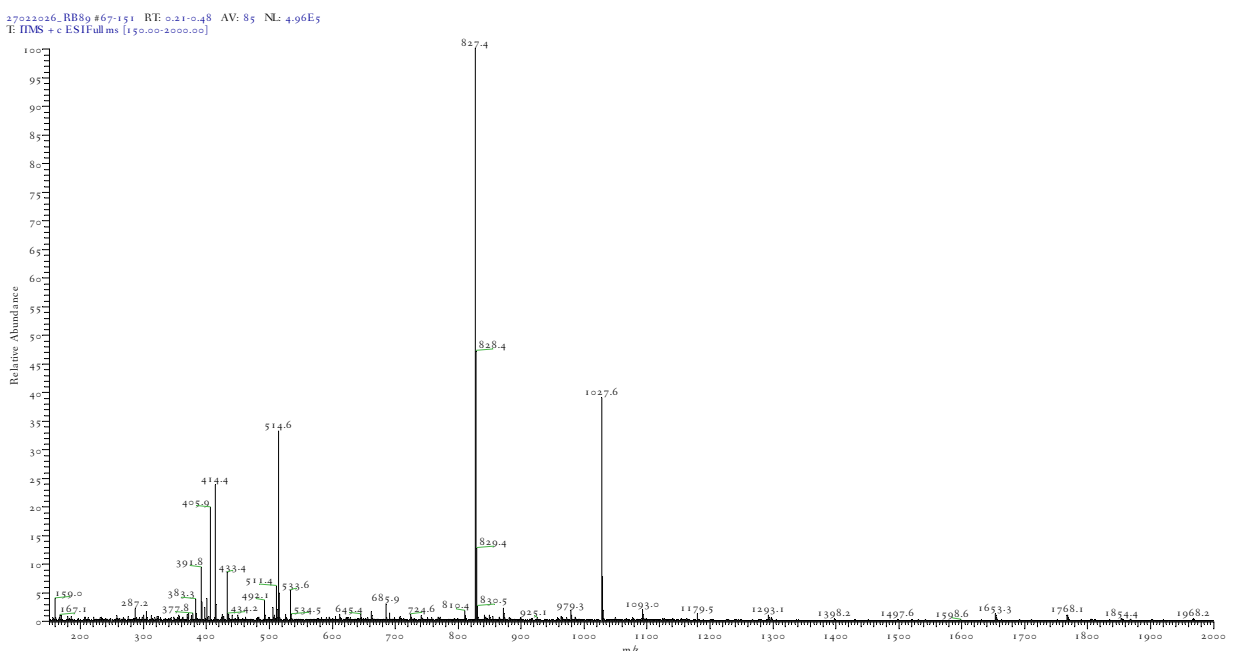

(F)

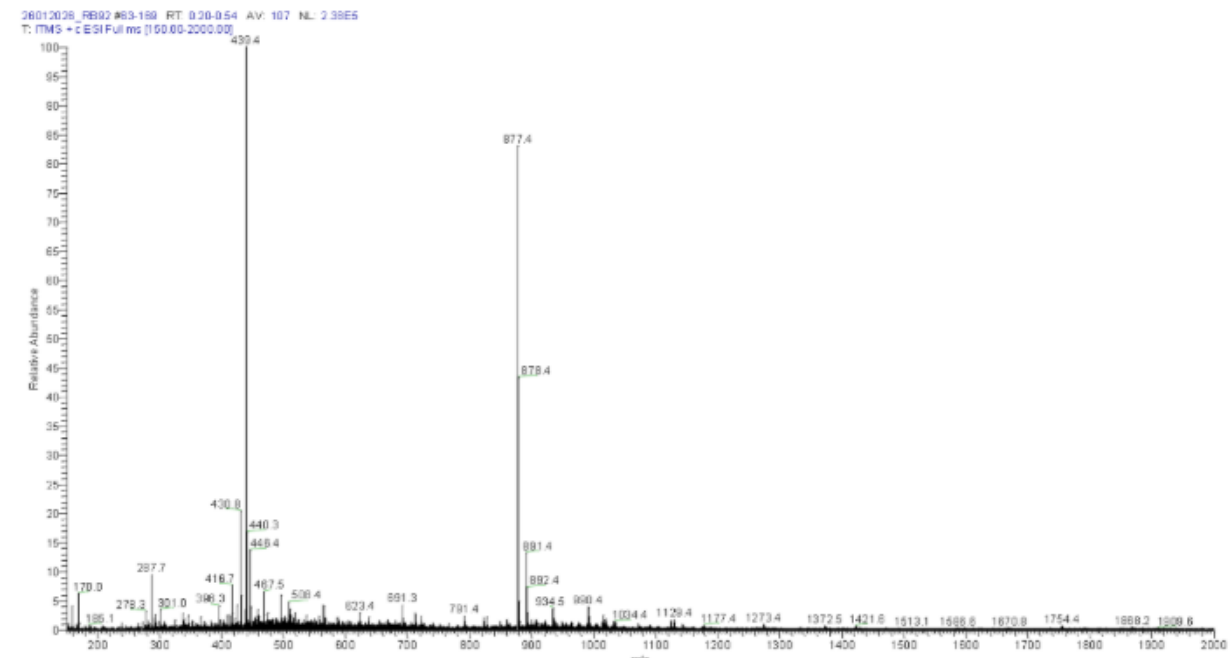

(G)

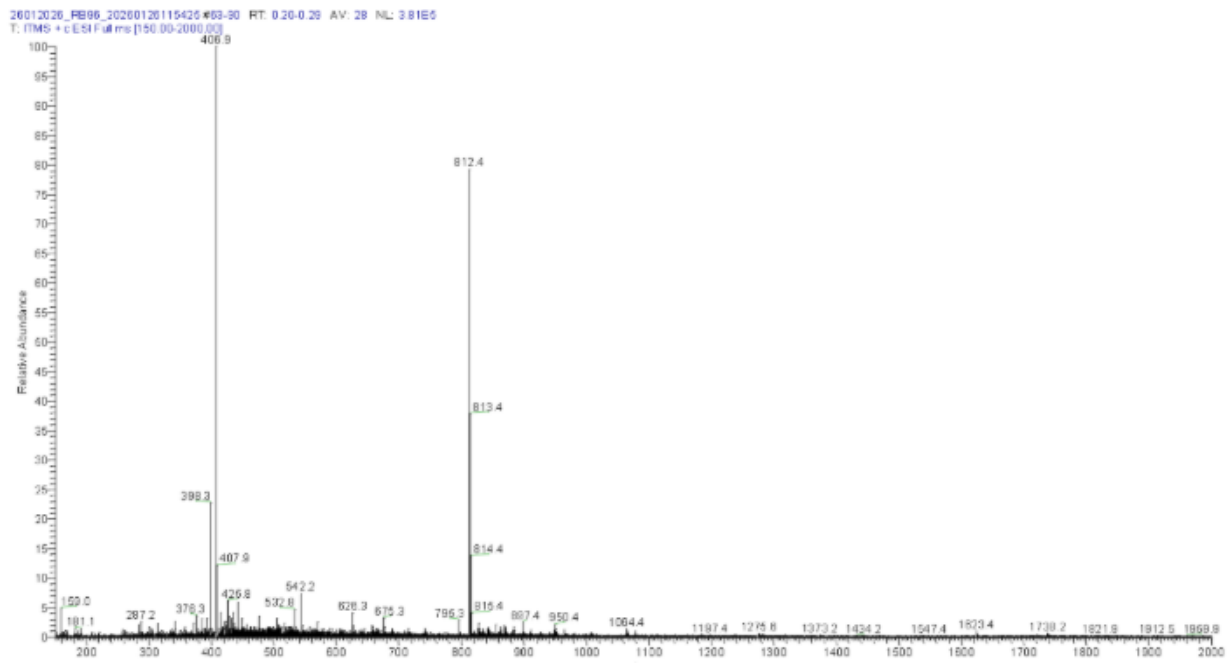

(H)

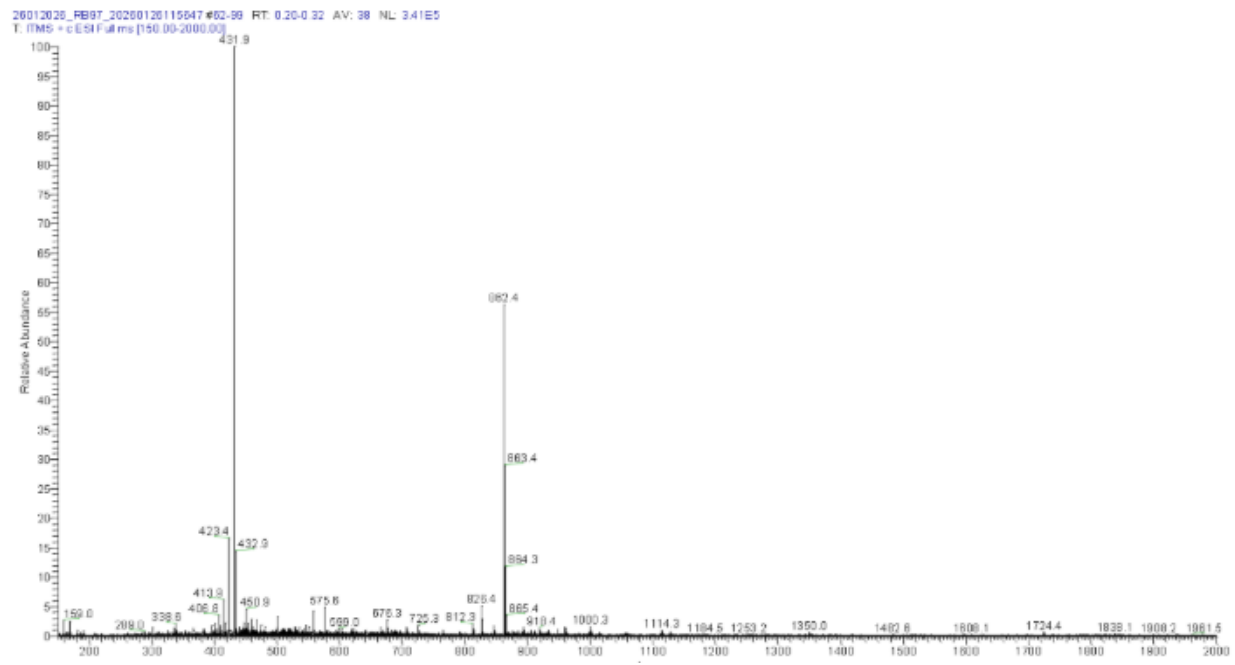

(I)

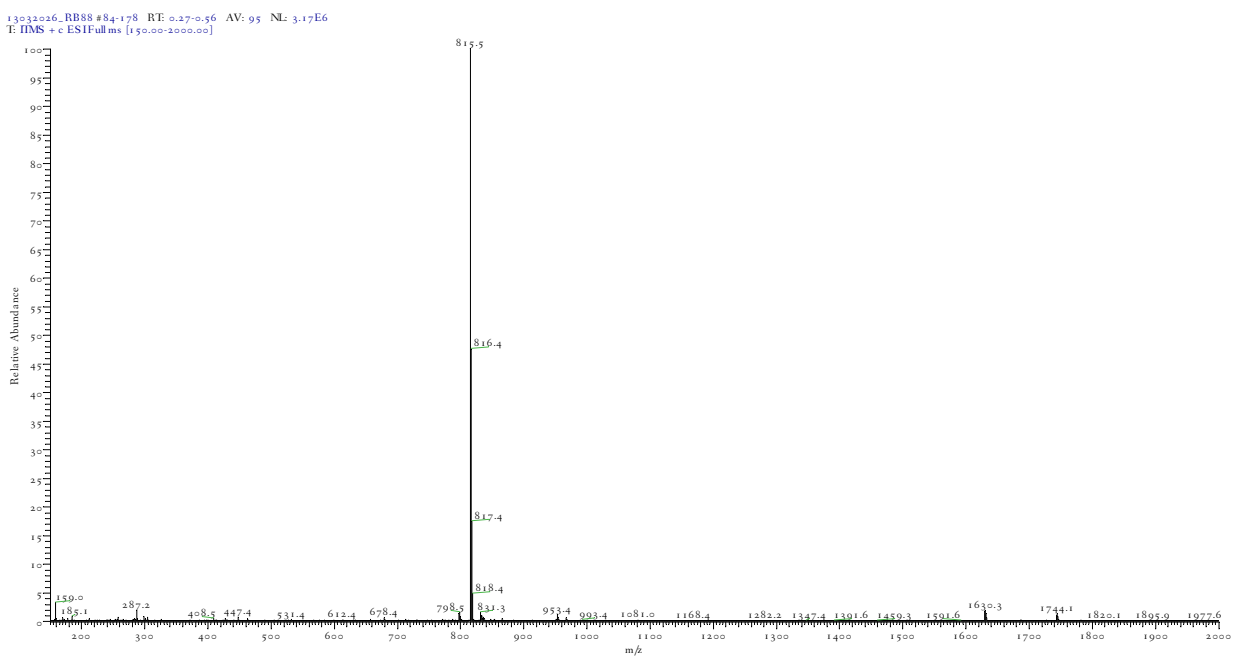

(J)

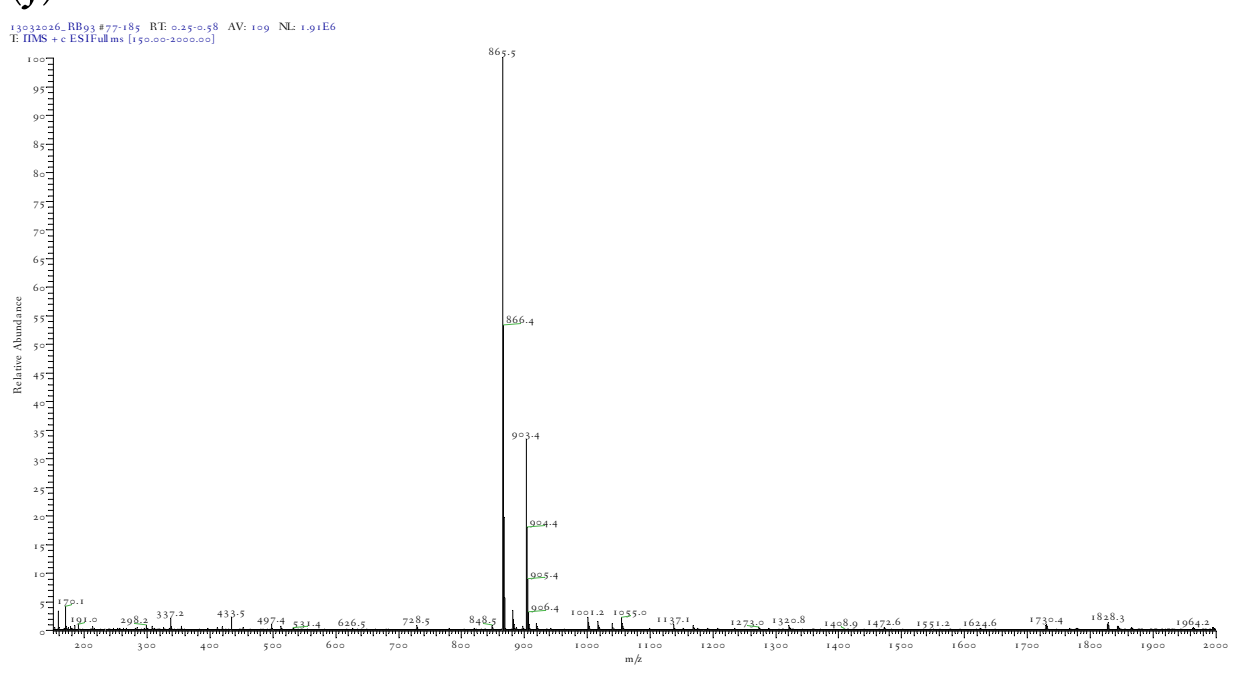

(K)

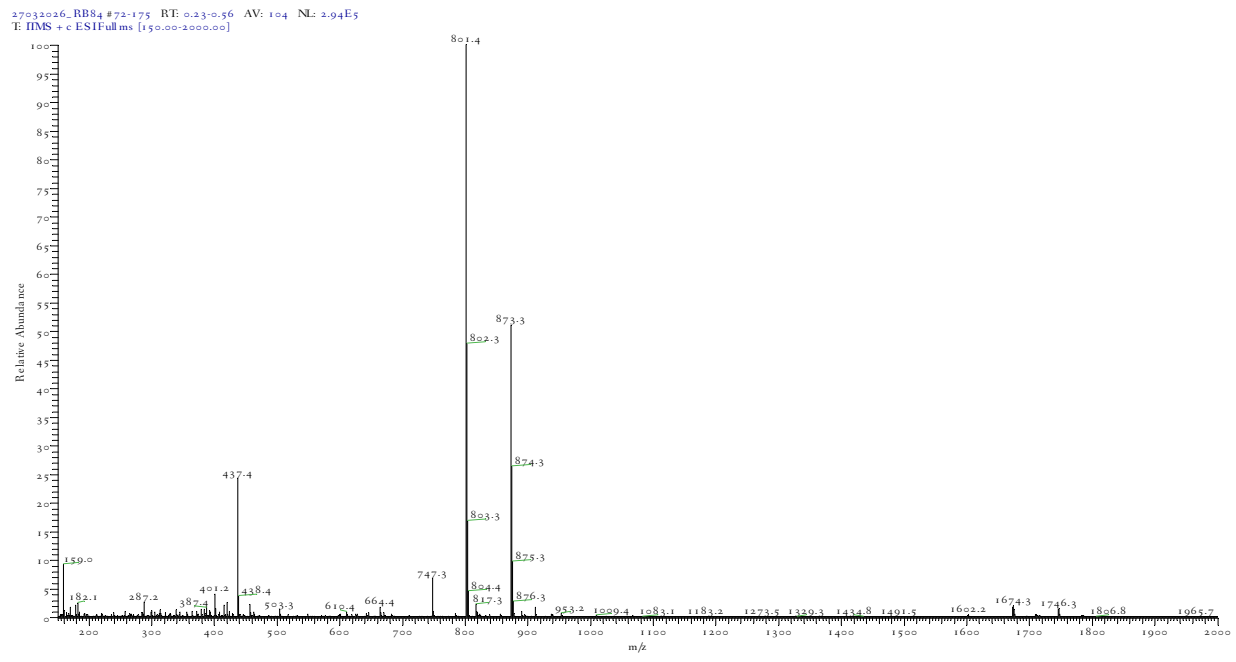

(L)

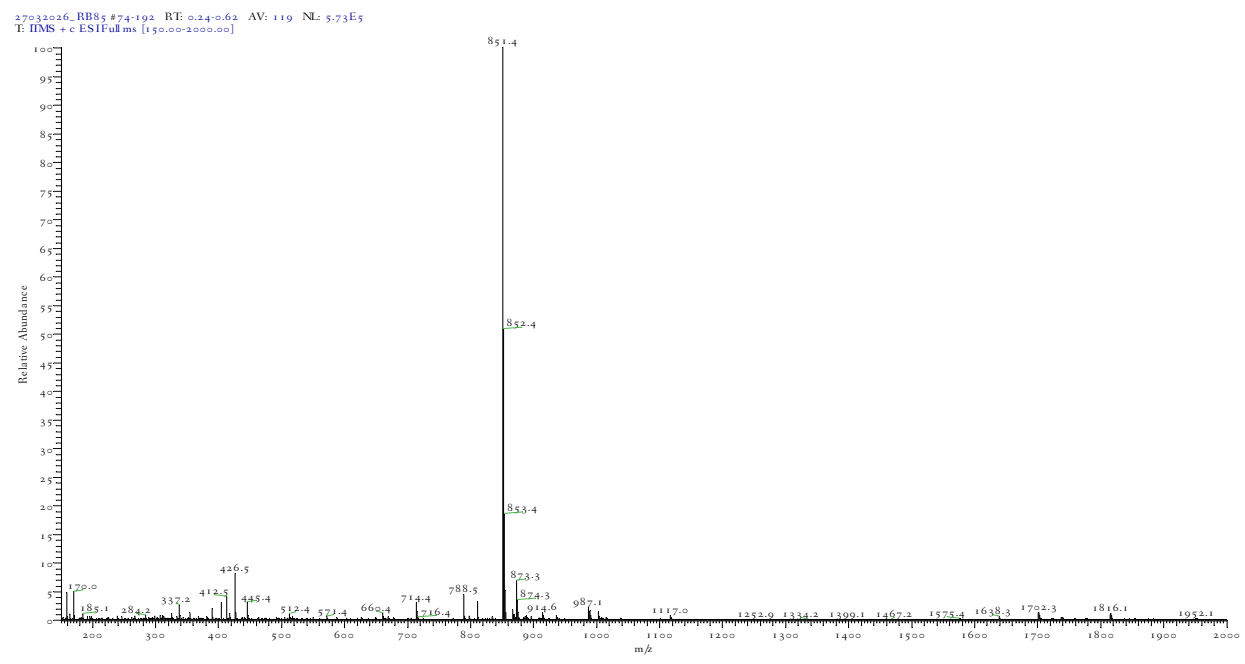

Figure S13. MS spectra for **RB-91** (**6**) (A), **RB-90** (**7**) (B), **RB-94** **(8)** (C), **RB-95** **(9)** (D), **RB-89** **(10)** (E), **RB-92** **(11)** (F), **RB-96** **(12)** (G), **RB-97** **(13)** (H), **RB-88** **(14)** (I), **RB-93** **(15)** (J), **RB-84** **(16)** (K), **RB-85** **(17)** (L).

4. **Table S2**. *Concentration–response profiles for cAMP accumulation.*

		Agonist activity									Antagonist activity						
Peptide identifier	Seq. #	EC_{50} hMC3R (nM)	pEC_{50} hMC3R	E_{MAX} hMC3R	ΔLog (E_{MAX}/EC_{50})	EC_{50} hMC4R (nM)	pEC_{50} hMC4R	E_{MAX} hMC4R	ΔLog (E_{MAX}/EC_{50})	ΔΔLog (E_{MAX}/EC_{50}) hMC4R/hMC3R selectivity	IC_{50} hMC3R (nM)	pIC_{50} hMC3R	Inhibition (%) hMC3R	IC_{50} hMC4R (nM)	pIC_{50} hMC4R	Inhibition (%) hMC4R	hMC4R/hMC3R fold selectivity[a]
**RB-91**	**6**	241	6.619	85.5	-2.226	4.2	8.373	98.0	-0.080	140	> 10,000	<5	0	7.2	8.143	18.0	
**RB-90**	**7**	470	6.328	5.4	-3.716	138	6.862	14.8	-2.415	20	> 10,000	<5	0	706	6.151	67.9	>100
**RB-94**	**8**	261	6.583	93.2	-2.223	19	7.732	102.3	-0.702	33	> 10,000	<5	0	33	7.478	15.2	
**RB-95**	**9**	168	6.774	6.2	-3.210	63	7.200	21.8	-1.907	20	1,809	5.743	46.2	185	6.733	65.0	10
**RB-89**	**10**	133	6.876	97.8	-1.910	8.5	8.073	105.1	-0.350	36	>10,000	<5	0	16	7.785	12.8	
**RB-92**	**11**	136	6.867	11.8	-2.837	108	6.965	58.0	-1.715	13	2,074	5.683	56.7	97	7.015	29.3	
**RB-96**	**12**	2,420	5.616	101.9	-3.152	115	6.938	96.8	-1.520	43	>10,000	<5	0	217	6.663	7.7	
**RB-97**	**13**	148	6.831	85.8	-2.013	16	7.805	92.7	-0.674	22	306	6.514	2.8	85	7.073	7.5	
**RB-88**	**14**	10	8.016	94.9	-0.783	0.28	9.553	108.3	1.142	84	>10,000	<5	0	0.40	9.397	17.4	
**RB-93**	**15**	19	7.723	9.0	-2.100	3.3	8.480	21.9	-0.624	30	697	6.157	64.8	15	7.828	65.7	47
**RB-84**	**16**	207	6.684	84.0	-2.168	20	7.692	97.2	-0.765	25	>10,000	<5	3.5	60	7.225	13.3	
**RB-85**	**17**	271	6.568	9.7	-3.222	62	7.207	16.2	-2.030	16	>10,000	<5	0	316	6.501	78.4	>100

[a] Only for peptides with Inhibition (%) > 50 for hMC4R, and ≥5-fold shifts in IC_{50} values from hMC4R over hMC3R.

5. Predicted conformation of peptide 6 (RB-91)

Conformational prediction for peptide **6** was performed using the Rosetta software suite, version 3.15 (RosettaCommons, University of Washington, USA). An initial sampling of 100 attempts yielded 71 valid structures, with *RB91_0012* showing the lowest Rosetta score (3.13715 REU). An independent extended sampling of 1000 attempts generated 729 valid structures, which were clustered based on the backbone N–Cα–C atoms of all six residues using a 1.0 Å RMSD cutoff. Thirty clusters were identified, with the most populated containing 254 structures (34.8%). A representative structure from this cluster *(RB91_0635)* showed a backbone RMSD of 0.714 Å relative to *RB91_0012*, indicating convergence of the independent sampling runs toward a recurrent low-energy conformational family. Structural analysis of this family revealed a type II-like β-turn involving Dab^1-His^2-DPhe^3-Arg^4 (Figure S14).

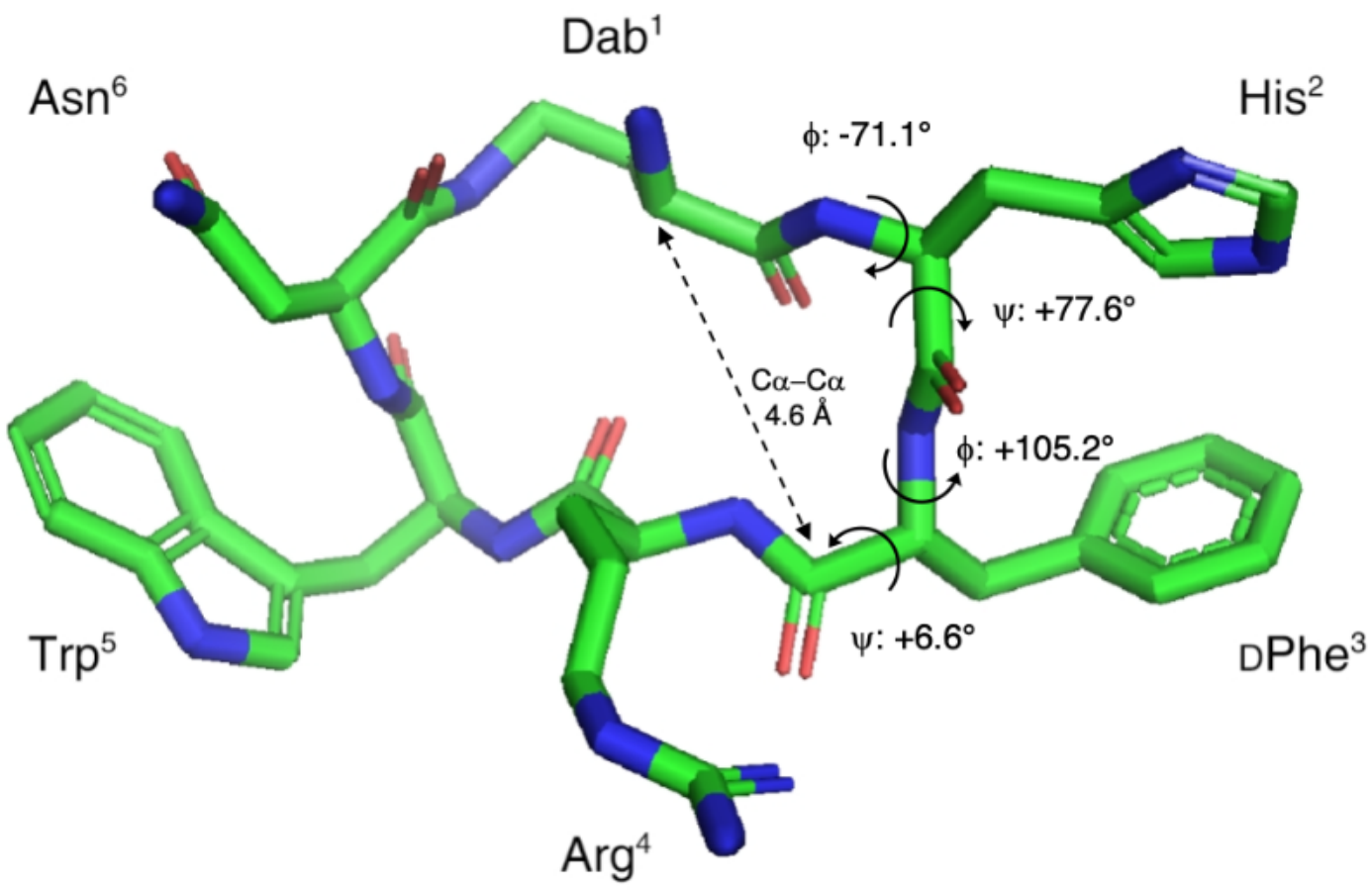

Figure S14. RosettaCommons-predicted conformation of peptide **6** (**RB-91**), showing the type II-like β-turn involving the Dab^1-His^2-DPhe^3-Arg^4 region. The displayed conformer represents the most populated conformational cluster obtained from the extended sampling.
